# The Validity of the 2-Point Method for Assessing the Force-Velocity Relationship of the Knee Flexors and Knee Extensors: The Relevance of Distant Force-Velocity Testing

**DOI:** 10.3389/fphys.2022.849275

**Published:** 2022-06-24

**Authors:** Matic Sašek, Dragan M. Mirkov, Vedran Hadžić, Nejc Šarabon

**Affiliations:** ^1^ Faculty of Health Sciences, University of Primorska, Izola, Slovenia; ^2^ InnoRenew CoE, Izola, Slovenia; ^3^ Faculty of Sport and Physical Education, University of Belgrade, Belgrade, Serbia; ^4^ Faculty of Sport, University of Ljubljana, Ljubljana, Slovenia; ^5^ Andrej Marušič Institute, University of Primorska, Koper, Slovenia; ^6^ S2P, Science to Practice, Ltd., Ljubljana, Slovenia

**Keywords:** force, velocity, isokinetic, single-joint, 2-point, validity

## Abstract

Over the past decade, force-velocity (F-v) profiling has emerged as a promising tool for assessing neuromuscular capacity to design individually tailored interventions in diverse populations. To date, a limited number of studies have addressed the optimization of the linear method for measuring F-v profiles of single-joint isokinetic movements. We aimed to simplify the measurement of knee extension (KE) and knee flexion (KF) isokinetic tasks by evaluating the most appropriate combination of two velocities (i.e., the 2-point method). Twenty-two healthy participants (11 males and 11 females) were included in the study. Isokinetic peak torque was measured at nine angular velocities (30-60-90-120-150-180-210-240-300°/s) and under isometric conditions (at 150° and 120° of KF for KE, and KF, respectively). Maximal theoretical force (F_0_), maximal theoretical velocity (v_0_), slope of the relationship (S_fv_) and maximal theoretical power (P_max_) were derived from the linear F-v profiles of KE and KF and compared between the 9-point method and all possible combinations (36 in total) of the 2-point methods. The F-v profiles obtained from nine points were linear for KE (R2 = 0.95; 95% CI = 0.94–0.96) and KF (R2 = 0.93; 95% CI = 0.90–0.95), with F_0_ underestimating isometric force. Further analyses revealed great to excellent validity (range: ICCs = 0.89–0.99; CV = 2.54%–4.34%) and trivial systematic error (range: ES = −0.11–0.24) of the KE 2-point method when force from distant velocities (30°/s, 60°/s or 90°/s combined with 210°/s, 240°/s or 300°/s) was used. Similarly, great to excellent validity and trivial systematic error of the KF 2-point method for F0 and Pmax (range: ICC = 0.90–0.96; CV = 2.94%–6.38%; ES = −0.07–0.14) were observed when using the previously described combinations of velocities. These results suggest that practitioners should consider using more distant velocities when performing simplified isokinetic 2-point single-joint F-v profiling. Furthermore, the F-v profile has the potential to differentiate between the mechanical properties of knee extensors and flexors and could therefore serve as a potential descriptor of performance.

## 1 Introduction

Sport performance levels are highly dependent on mechanical efficiency, described as the ability to maximize external force, velocity, and external power production during a given motor task (López-Segovia et al., 2011; Seiler et al., 2019). The relationship between these external mechanical characteristics is described by the force-velocity (F-v) relationship or the so-called F-v profile, which has been shown to be approximately linear for multi-joint tasks (Samozino et al., 2008; Jaric, 2016). Therefore, a linear regression is used to calculate the F-v profile parameters, maximal external theoretical force (F_0_), maximal theoretical velocity (v_0_), slope of the F-v relationship (S_fv_), and maximal external theoretical power (P_max_), reflecting the neuromuscular capacity of the tested muscle groups (Morales-Artacho et al., 2018). Optimal relationship between force and velocity, expressed as S_fv_ was found to be important for maximizing performance. Namely, based on their F-v profile the athletes capable to exert relatively high force at low velocity can be described as force-dominant, while the ones producing relatively low force at high velocity can be described as velocity-dominant (Lindberg et al., 2021). However, the definition of the optimal profile, especially for different motor tasks such as single-joint movements, represents a challenging task. Some indices suggest that the optimal F-v profile depends highly on the movement task (Samozino et al., 2012). Besides the necessity to describe mechanical capacity of muscles, particularly important could be the information regarding potential discrepancies between optimal F-v profile of the task and individual F-v profile indicating the imbalances in external mechanics. Moreover, individually tailored training interventions based on profile optimization were shown to be highly effective and significantly increased jumping and sprinting performance (Baena-raya et al., 2021a).

With this in mind, it is not surprising that F-v profiles have attracted considerable interest in the last decade among researchers and practitioners who aimed to optimize and suit the measurement profile to various motor tasks. In particular, high linearity of the F-v relationship has been observed in multi-joint tasks such as vertical jumps, squats or bench press (Samozino et al., 2008; Samozino et al., 2012; Jaric, 2015; García-Ramos et al., 2016; Jiménez-Reyes et al., 2016; García-Ramos et al., 2021). On the other hand, only a few studies have considered the F-v or torque-velocity profiles (i.e., reporting maximal theoretical values at zero force/torque or zero velocity and the slope of relationship) of single-joint movements isokinetic conditions (Lemaire et al., 2014; Grbic et al., 2017a; Janicijevic et al., 2019). This can be explained by the well-known observations of Hill (1938), who found a hyperbolic intra-muscular relationship between force and v, which devalued the use of the linear F-v profiling method from the outset. Since then, various deviations of the F-v relationship from the double-hyperbolic to the quasilinear relationship have been observed in *in-vivo* studies investigating the external mechanics of isokinetic single-joint tasks (Wickiewicz et al., 1984; Harris and Dudley, 1994; Finni et al., 2003), focusing mainly on the force at one of the ends of the F-v curve (i.e., at high forces and low velocities), where the largest deviation from a linear relationship has been described (Seger and Thorstensson, 2000). Nevertheless, some studies observed the linear F-v relationship (R^2^ > 0.96) of knee extension (KE) and knee flexion (KF) tasks in the range of angular velocities from 30° to 240°/s (Lemaire et al., 2014; Grbic et al., 2017b; Janicijevic et al., 2019). These observations, which allow F-v profiling and extrapolation of F_0_ and v_0_ outcomes, could potentially be of great importance and practical value. Detailed information on the F-v profile of specific muscle groups could allow the exploration of novel parameters describing between-muscular ratios [e.g., quadriceps to hamstring (H/Q) ratio] and could be interesting to further distinguish muscular function imbalances. In addition, individualization of strength training performed at mid-range velocity based on the F-v profile would be possible.

Although F-v profiling protocols obtained from multiple loads are reliable and valid, they are time-consuming and could lead to muscle fatigue (Garcia-Ramos and Jaric, 2018). As a solution, Jaric (2016) proposed an optimized profiling method using only two loads (2-point method). Since then, different authors have confirmed the validity and reliability of the 2-point method in multi-joint tasks (Garcia-Ramos and Jaric, 2018; Janicijevic et al., 2020) and single-joint tasks (Grbic et al., 2017b; Janicijevic et al., 2019). Perez Castilla et al. (2018) observed the highest agreement between the two- and multi-point methods in the bench press throw when two very distant loads (20 and 70% 1RM) were used. Similar findings were observed for vertical jumps by Garcia-Ramos and Jaric (2018), who suggested the use of the two most distant loads (i.e., 0 and 75 kg) when evaluating the F-v relationship in vertical jumps. For a single-joint task, the agreement between two- and multi-point methods was assessed only with the combination of data from two most commonly used velocities, 60°/s and 180°/s. The results indicated high agreement and association between all F-v parameters in study by Grbic et al. (2017a), where high validity of the 2-point method was observed only for F_0_ in the study by Janicijevic et al. (2019). Although it seems that 60°/s and 180°/s method is valid, previous studies investigating 2-point method validity on multi-joint tasks emphasized the use of more distant velocity points. Those could yield even higher agreement in single-joint tasks, due to a lower tendency to the erroneous prediction of end values.

Isokinetic testing is commonly used to assess lower-limb strength deficits. For example, the relationship between quadriceps and hamstring concentric strength is used to assess knee function. At lower angular velocities, quadriceps exert a relatively greater torque than hamstring (at 12°/s H/Q ratio = 0.52), and with increasing velocity the strength equalizes, reaching an H/Q ratio of 0.80 at 300°/s (Baroni et al., 2020). Furthermore, part of the literature suggested asymmetry in strength at high and low velocities as an important risk factor for hamstring and anterior cruciate ligament injuries (Myer et al., 2009; Lee et al., 2018), while some studies neglected these assumptions (van Dyk et al., 2016; Kellis et al., 2019). A comparison of the F-v profile ratios in addition to the strength ratios between quadriceps and hamstrings would provide unique insight into the differences in mechanical performance. An important feature of the F-v profile is that it describes the relationship between force or power and velocity across the velocity spectrum, which could be an important risk factor for injury during rapid movements, as simultaneous action of the quadriceps and hamstring muscles at different velocities is important for normal knee function. However, the sensitivity of the F-v profile to detect differences between muscle groups must be evaluated first. To our knowledge, only Janicijevic et al. (2019) had already assessed differences between four muscle groups (knee extensors, knee flexors, elbow flexors and elbow extensors) and concluded that the F-v profile is muscle and task-specific. Further evidence is therefore needed to investigate the sensibility of the F-v profile for various single-joint movements.

For this purpose, we evaluated the F-v profile of KE and KF using nine different velocities (9-point method) and all possible combinations of two velocities (2-point method) together with the isometric strength at the angle of optimal KE and KF length-force relationship. At the preliminary level, we aimed to evaluate differences 1) in the degree of fit between the linear and polynomial multi-point methods and 2) in maximal theoretical force between both calculation methods (linear F_0_ and polynomial F_0_) and the isometric method. Secondarily, we 3) aimed to investigate the concurrent validity of 36 2-point methods with respect to the 9-point method. Finally, we 4) assessed the sensibility of F-v parameters to discriminate between the mechanical capacity of KE and KF. Based on the results of previous studies, we hypothesized to obtain the following: 1) high association and agreement between linear and polynomial 9-point methods with potentially 2) underestimated isometric force with F_0_, 3) significant differences between all F-v parameters between KF and KE, and 4) the highest degree of agreement in the F-v profile between the linear 9-point and 2-point method that used the most distant force and velocity points.

## 2 Materials and Methods

### 2.1 Participants

Eleven male and eleven female kinesiology students (23.3 ± 3.8 years, 1.73 ± 0.10 m, and 63.1 ± 4.4 kg) voluntarily participated in this study. All participants were physically active (minimal 3 to 6 aerobic and/or strength training sessions per week in the last year) and had no back or lower limb injuries or pain in the past 6 months. In addition, all participants had previous experience with isokinetic testing or training. Prior to the experiment, participants were informed of the aims and procedures of the study. An informed consent form was provided and approved by the Institution’s Ethics Committee (approval number: 0120-99/2018/5). The study was conducted in accordance with the Declaration of Helsinki.

### 2.2 Study Design

**FIGURE 1:**
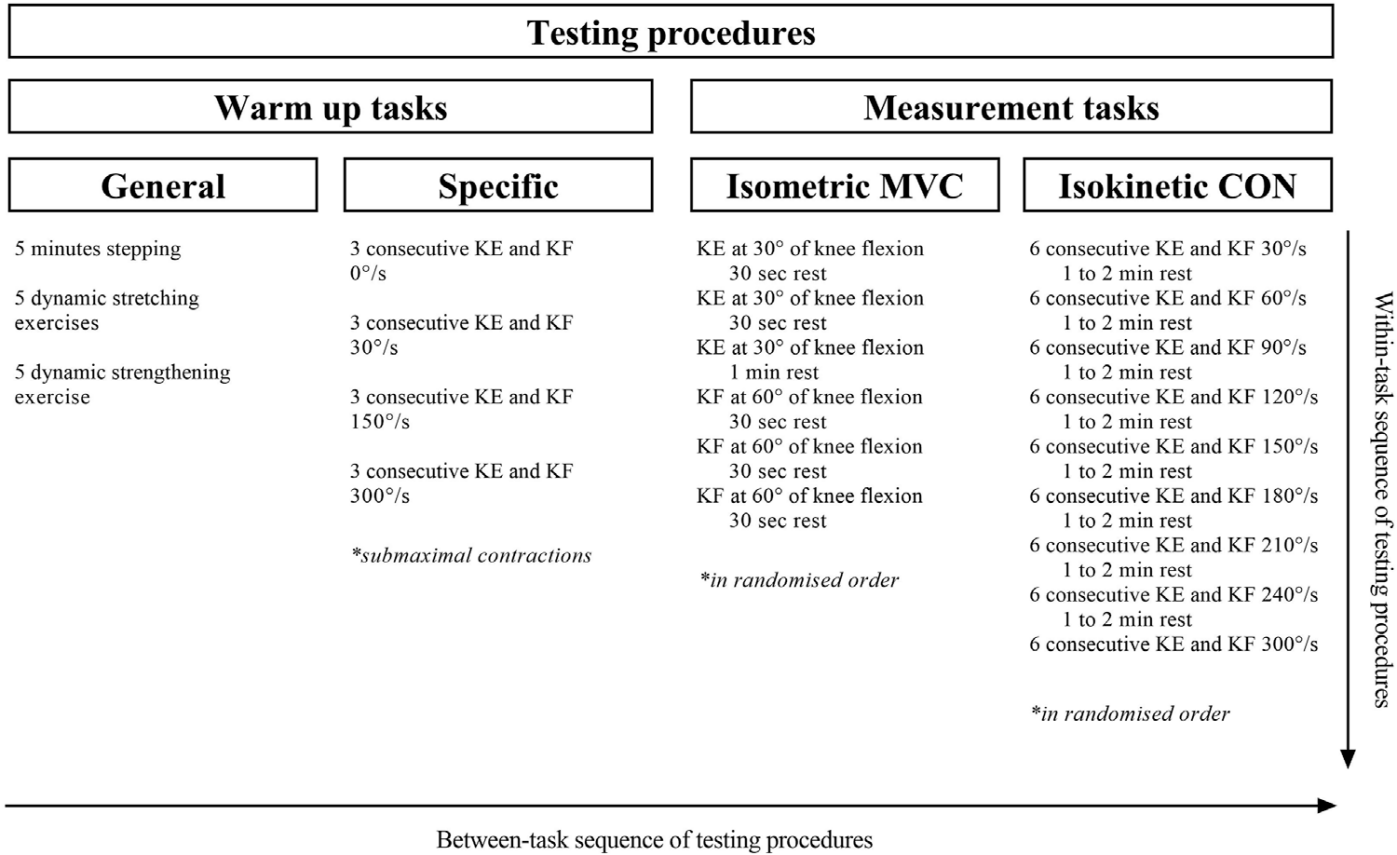
Graphical presentation of experimental design and measurement procedures of the study. KF, knee flexion; KE, knee extension; F, force; ISO, isometric; POLY, polynomial; LIN, linear; F_0_, maximal theoretical force; V, velocity; N, Newtons; kg, kilograms; m, meters; s, seconds.

For the purpose of the study, we used a cross-sectional design to examine the concurrent validity of the linear and 2-point methods for calculating the characteristics of the F-v relationship of isokinetic KE and KF (see Figure 1 for details). Each participant reported for testing for an approximately 1-h session consisting of a standardized warm-up and measurement protocol.

### 2.3 Testing Procedures

All measurements were performed by an experienced examiner on a Humac NORM isokinetic dynamometer (CSMi, Stoughton, Massachusetts) in the laboratory. The warm-up included 5 min of stepping (100 bpm) on a 20-cm box and 5 dynamic stretching and 5 strengthening lower-limb exercises. Afterwards, the participants were seated in an upright position with 100° seat tilt and fixed with straps around the chest, pelvis, thigh, and ankle. The axis of rotation of the knee was aligned with the femoral condyle and the lever length was set at the end of the tibia (with maximally dorsiflexed ankle) and measured for the purpose of further analyzes. Prior to measurement, a demonstration and explanation of the protocol were given, as well as a specific warm-up, which included 3 consecutive repetitions of KE and KF at 0°/s, 30°/s, 150°/s, and 300°/s.

The strength tests were performed under isometric and isokinetic conditions. The isometric tests were performed at 60° and at 30° of KF to assess the strength of KE and KF, respectively (where 0° corresponds to full extension). Participants were verbally instructed before the test to “gradually increase the level of KF/KE and maintain maximum exertion for at least 3 s,” therefore, the contraction lasted ∼5 s in total. Three consecutive repetitions were performed with 30 s rest between trials and 1 min rest between the tasks. The isokinetic concentric strength of KE and KF was tested between 90° and 170° of knee extension at 9 angular velocities (30, 60, 90, 120, 150, 180, 210, 240, and 300°/s) to assess moment torque production capacity across a wide range of angular velocities. During the test, 6 KE and KF repetitions were performed in a sequence at each angular velocity. The last four repetitions were considered in further analyzes. The tests were performed in randomized order, with 1–2 min rest between trials. Verbal encouragement and real-time visual feedback of the torque-velocity curves during testing were provided (Hald and Bottjen, 1987).

### 2.4 Data Acquisition

Torque-time data were sampled at 500 Hz and low-pass filtered using a second-order Butterworth filter (5 Hz). For further analysis, the trial with the highest peak torque in the isokinetic part of the torque-time curve was used. Peak torques and angular velocities were converted to Force (F) and linear velocity (v) by dividing or multiplying measured values by the subject’s lever arm length. Then, to account for interindividual differences, the froce was normalized to body size (N/kg^2/3^) as suggested by Jaric (2002). The F-v relationship and F-v profile were calculated using a polynomial and a linear regression line fitted to the force at nine different velocities (9-point polynomial or linear method). Next, a linear regression line was fitted to the force at all possible (36) combinations of two velocities (2-point method). F_0_ and v_0_ of KE and KF were calculated with all methods by extrapolating the regression line to its limits (i.e., zero force and zero velocity). Finally, the slope of the relationship (S_Fv_) and maximal theoretical external power production (P_max_) were calculated as follows (Eqs 1, 2):

$$S_{Fv}=\;\frac{F_{0}}{v_{0}}\;$$ (1)

$$P_{max}=\;\frac{F_{0}\times v_{0}}{4}\;$$ (2)

In order to assess the deviations in the calculation of maximal force production capacity using the theoretical (polynomial and linear 9-point) method, KE and KF peak isometric torque was also divided by lever length and normalized per body size (N⋅kg^2/3^).

### 2.5 Statistical Analysis

Statistical analyzes were performed using SPSS Statistics (version 26.0, Armonk, NY, IBM Corporation) and Prism software (version 9.0.2, San Diego, CA, GraphPad Software). Descriptive statistics were calculated and reported as mean ± standard deviation. The normality of data distribution was tested using the Shapiro-Wilk test. When the assumption of normality has been violated, nonparametric tests or nonparametric data were used. In the preliminary phase of the analyzes, the fit of linear and polynomial methods to the F-v data was compared using the paired-sample t-test. Next, paired-sample *t*-test was used together with Cohen’s d effect size coefficient (ES), and Pearson’s r correlation coefficient to assess between-method (linear and polynomial in regard to isometric) differences in F_0,_ and between-task (KE in regard to KF) differences in the F-v profile. The criteria for interpreting the magnitude of the ES were as follows: negligible (< 0.20), small (0.20–0.50), moderate (0.50–0.80), and large (> 0.80) (Cohen, 1988); whereas Pearson’s r was interpreted as weak (0.00–0.30), moderate (0.31–0.50), strong (0.51–0.70), very strong (0.71–0.99), or perfect (1.00) (Hopkins et al., 2009)*.*

In the consequent phase of analysis, the concurrent validity of the linear 2-point method in regard to the 9-point linear method was tested in multiple ways. The paired-sample t-test was used to assess the agreement between the methods, coefficient of variation [CV (%) = SEM/mean x 100] to examine within-individual error, and two-way mixed average and single intra-class correlation coefficients (ICC_s_ and ICC_a_) with respective 95% confidence intervals to quantitatively interpret the agreement between the methods. ICC was considered fair (0.40–0.59), moderate (0.60–0.74), good (0.75–0.90), or excellent (> 0.90) (Koo and Li, 2016). The most valid 2-point methods (e.g., those that showed good to excellent agreement (ICCs lower bound of 95% CI ≥ 0.75), no systematic bias (*p* ≥ 0.05), and low within-individual error (CV lower bound of 95% ≤ 10%) separately for F_0_, v_0_, S_fv_, and P_max_) were selected and presented as Bland-Altman plots to visually elucidate the agreement.

## 3 Results

A normal distribution was found for all linear and polynomial F-v profile parameters of the KE and KF 9-point methods (all p’s > 0.076). However, the intra-individual fit of the regression methods to the F-v data was not normally distributed. Therefore, the correlation coefficients were converted to nonparametric z' values using Fisher z-transformation, and the assumption of normality of distribution was met (all p’s > 0.258).

### 3.1 Linearity of Force-Velocity Relationship and Maximal Isometric Force Estimation

**FIGURE 2:**
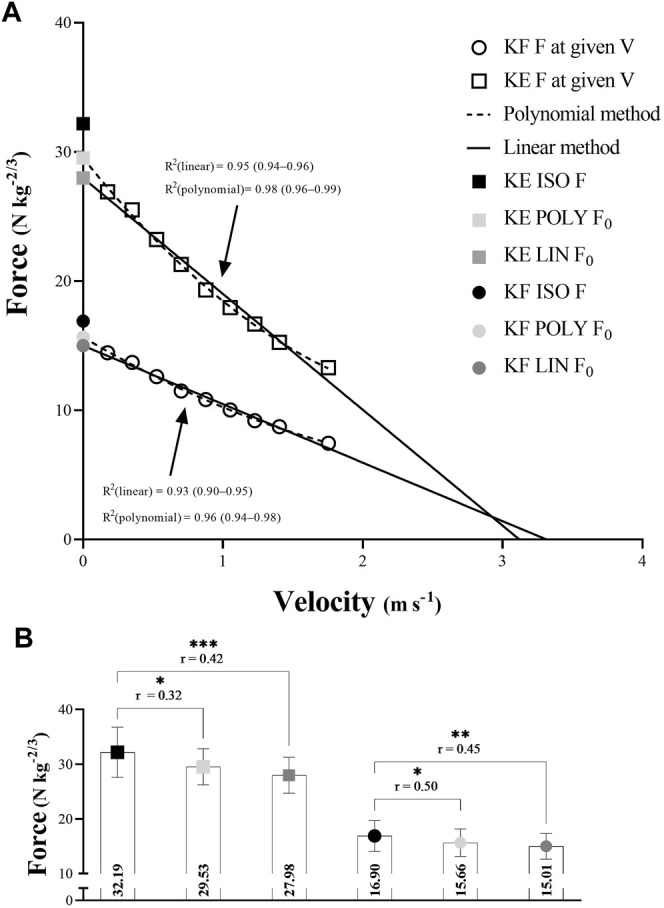
**(A)** Polynomial and linear model with respective isometric force and **(B)** the differences between polynomial and linear maximal theoretical force and maximal isometric force. KF, knee flexion; KE, knee extension; F, force; F_0_, maximal theoretical force; V, velocity; ISO, isometric; POLY, polynomial; LIN, linear; N, Newtons; kg, kilograms; m, meters; s, seconds. *p < 0.05, **p < 0.01, ***p < 0.001.

The differences in the fit of the polynomial and linear regression methods to the F-v data are shown in Table 1. Although the values presented in Figure 2 section A showed a near-perfect fit of both regression models for KE and KF (all R^2^ > 0.93), the polynomial fit was significantly better for both movement tasks (ES = −1.13 and ES = −0.82, respectively). Regardless of the differences in fit, both polynomial and linear method F_0_ significantly underestimated maximal isometric KE force and KF froce (see section B at Figure 2). Additionaly, a moderate association between KE F_0_ and isometric force (*r* = 0.32 for the polynomial; *r* = 0.42 for the linear), and KF F_0_ and isometric force (*r* = 0.50 for the polynomial; *r* = 0.45 for the linear) was observed.

**TABLE 1:** Differences between the fit of 9-point methods to the KE and KF F-v data.

	Mean (±SD)			Paired sample *t*-test			
		Polynomial	Linear	Diff (±SD)	t	p	ES
KE	z’	2.74 (0.49)	2.14 (0.43)	0.46 (0.41)	−5.26	<0.001	−1.13
	r	0.99 (0.01)	0.96 (0.02)				
KF	z’	2.53 (0.53)	2.28 (0.28)	0.40 (0.37)	−5.02	<0.001	−0.82
	r	0.98 (0.02)	0.98 (0.01)				

KE, knee extension; KF, knee flexion, p—*p* value; SD, standard deviation; ES, effect size.

### 3.2 Differences Between KF and KE F-v Profile

In general, significant differences in 9-point method F-v profile were found between KE and KF (see Table 2). In contras to ∼60% greater F_0_ and _∼_54% greater P_max_ of KE, KF possessed ∼6% larger v_0_ and ∼66% flatter S_fv_. Strong to very strong relationship between KE and KF was observed for all F-v parameters except for S_fv,_ where a weak correlation was observed.

**TABLE 2:** Differences between KE and KF F-v profiles.

F-v Profile	Mean (±SD)		Between-task differences		
	KE	KF	Diff (±SD)	t	r
F_0_ (N kg^−2/3^)	27.98 (3.28)	15.01 (2.35)	12.75 (3.75)***	22.10	0.56**
v_0_ (ms^−1^)	3.16 (0.53)	3.36 (0.59)	−0.2 (0.44)*	−2.13	0.71***
S_fv_ (Nms^−1^kg^−2/3^)	−9.06 (1.66)	−4.54 (0.74)	−4.51 (1.66)***	−12.72	0.22
P_max_ (W kg^−2/3^)	22.25 (5.33)	12.72 (3.46)	9.53 (3.03)***	14.72	0.85***

KE, knee extension; KF, knee flexion; F_0,_ maximal theoretical force; v_0_, maximal theoretical velocity; S_fv_, slope of force-velocity curve; P_max_, maximal theoretical power; t-t statistics; r, Pearson’s r correlation coefficient; SD, standard deviation. *p < 0.05, **p < 0.01, ***p < 0.001.

### 3.3 Validity of the 2-Point Method for Measuring F-v Profile

**FIGURE 3:**
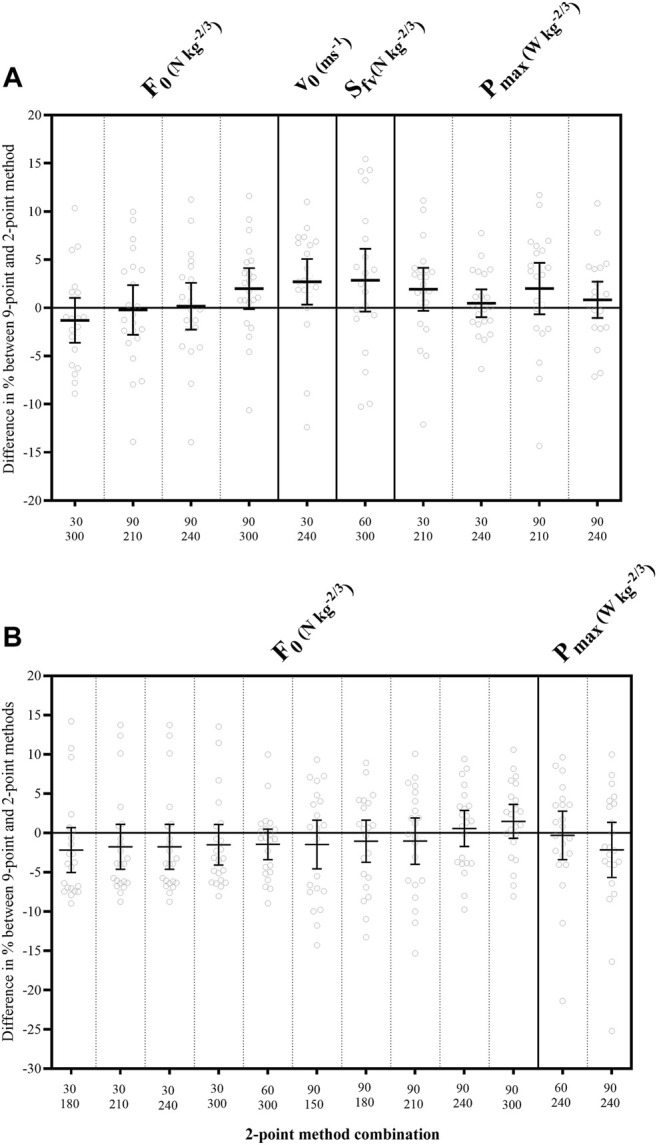
Bland—Altman statistics for combination of the 2-point methods with the highest validity. **(A)** graph presents knee extension and **(B)** graph presents knee flexion. Horizontal line shows mean bias—difference between the 2-point methods and 9-point method F-v parameters in %. Vertical red line shows 95% limits of agreement for bias. F_0_, maximal theoretical force; v_0_, maximal theoretical velocity; S_fv_, slope of force-velocity curve; P_max_, maximal theoretical power; N, Newtons; W, Watts; m, meters; s, seconds.

The validity of the 2-point methods for KE is shown in Table 3 and Figure 3 section A. Good to excellent agreement was found between the 9-point and specific 2-point methods for all F-v parameters. In particular, the combinations of low (from 30°/s to 90°/s) and high velocities (from 210°/s to 300°/s) gave no systematic error (all p’s > 0.06), negligible effect sizes (all ES’s < 0.17), the lowest within individual variation (all CV’s lower limit < 7.43%) and good to excellent agreement (all ICC_s_ lower limit > 0.75) with respect to the 9-point method.

**TABLE 3:** 2-point methods with the highest validity for KE task.

Variable	9p method	2p methods		Systematic error				Intraclass correlation		Within individual error		
	Mean (±SD)	COMB	Mean (±SD)	Bias (±SD)	t	p	ES	ICC_s_ (95% CI)	ICC_a_ (95% CI)	SEM	CV%	MDC
F_0_ (Nkg^−2/3^)	27.98 (3.28)	30–300	28.74 (2.98)	−0.35 (1.42)	−1.11	0.28	0.24	0.89 (0.75–0.95)	0.94 (0.86–0.98)	0.24	3.55 (2.70–5.19)	0.88
		90–210	28.13 (3.97)	−0.15 (1.72)	−0.41	0.69	0.04	0.89 (0.76–0.95)	0.94 (0.86–0.98)	0.37	4.33 (3.33–6.19)	1.01
		90–240	28 (3.84)	−0.02 (1.63)	−0.07	0.95	0.01	0.90 (0.77–0.96)	0.95 (0.87–0.98)	0.35	4.11 (3.17–5.88)	0.96
		90–300	27.48 (3.65)	0.5 (1.41)	1.67	0.11	−0.14	0.91 (0.80–0.96)	0.95 (0.89–0.98)	0.30	3.59 (2.76–5.13)	0.83
		60–180	29.31 (3.78)	−1.33 (1.13)	−5.51	<0.00	0.38	0.89 (0.25–0.97)	0.94 (0.4–0.98)	0.25	2.8 (2.15–3.99)	0.67
v_0_ (ms^−1^)	3.16 (0.53)	30–240	3.09 (0.64)	0.07 (0.19)	1.63	0.12	−0.11	0.94 (0.87–0.98)	0.97 (0.93–0.99)	0.04	4.33 (3.33–6.19)	0.11
		60–180	2.78 (0.53)	0.38 (0.31)	5.75	<0.01	−0.72	0.66 (−0.04–0.89)	0.80 (−0.07–0.94)	0.07	7.46 (5.74–10.66)	0.18
S_fv_ (Nms^−1^kg^−2/3^)	−9.06 (1.66)	60–300	−8.79 (1.53)	−0.27 (0.14)	−1.98	0.06	0.17	0.91 (0.79–0.96)	0.95 (0.88–0.98)	0.14	5.06 (3.89–7.22)	0.38
		60–180	−10.91 (2.44)	1.85 (0.29)	6.37	<0.01	−0.89	0.57 (−0.09–0.85)	0.73 (−0.19–0.92)	0.29	9.65 (7.43–13.79)	0.81
P_max_ (Wkg^−2/3^)	22.25 (5.33)	30–210	21.75 (4.93)	0.5 (1.35)	1.25	0.22	−0.06	0.97 (0.93–0.99)	0.99 (0.97–0.99)	0.28	4.18 (3.21–5.97)	0.77
		30–240	22 (5.01)	0.25 (1.03)	0.79	0.44	−0.03	0.99 (0.97–1.00)	0.99 (0.99–1.00)	0.17	2.54 (1.95–3.63)	0.47
		90–210	22.29 (5.41)	−0.04 (1.37)	1.73	0.10	−0.10	0.96 (0.91–0.98)	0.98 (0.95–0.99)	0.29	4.34 (3.34–6.21)	0.80
		90–240	22.57 (5.16)	−0.32 (1.33)	1.12	0.27	−0.05	0.98 (0.95–0.99)	0.99 (0.98–1.00)	0.22	3.28 (2.53–4.69)	0.61
		60–180	20.42 (5.11)	1.83 (1.66)	5.16	<0.01	−0.35	0.90 (0.34–0.97)	0.95 (0.50–0.99)	0.35	5.5 (4.23–7.86)	0.98

9p method, 9-point method; 2p method, 2-point method; COMB, a, combination of 2-point method; F0, maximal theoretical force; v_0_, maximal theoretical velocity; S_fv_, slope of force-velocity curve; P_max_, maximal theoretical power; ICCs, single intraclass correlation; ICCa, average intraclass correlation; t, t statistics; SEM, standard error of mean; ES, Cohen’s d coefficient; CV, coefficient of variation; SD, standard deviation; 95% CI, 95% confidence interval; MDC, minimal detectable change.

Similar observations were made for KF (see Table 4 and Figure 3 section B). The particular combinations 30-180, 30-210, 30-240, 30-300, 60-240, 60-300, 90-150, 90-180, 90-210, 90-240, and 90–300 showed little variation within individuals (all CV’s < 5.05%), good to excellent agreement (all ICC_s_ > 0.90), no systematic errors (all p’s > 0.06), and negligible effect size (all ES’s < 0.14) for F_0_ and P_max_ in respect to 9-point method. However, none of the 2-point methods provided sufficient validity for the v_0_ and S_fv_.

**TABLE 4:** 2-point methods with the highest validity for KF task.

Variable	9p method	2p methods		Systematic error				Intraclass correlation		Within individual error		
	Mean (±SD)	COMB	Mean (±SD)	Bias (±SD)	t	p	ES	ICCs (95% CI)	ICC_a_ (95% CI)	SEM	CV%	MDC
F0 (Nkg−2/3)	15.01 (2.35)	30–180	15.34 (2.48)	−0.34 (1.08)	−1.46	0.16	0.14	0.90 (0.77–0.96)	0.95 (0.87–0.98)	0.23	5.05 (3.88–7.21)	0.64
		30–210	15.33 (2.41)	−0.33 (1.00)	−1.53	0.14	0.14	0.91 (0.79–0.96)	0.95 (0.88–0.98)	0.21	4.66 (3.59–6.67)	0.59
		30–240	15.28 (2.44)	−0.27 (0.99)	−1.28	0.21	0.11	0.91 (0.80–0.96)	0.95 (0.89–0.98)	0.21	4.63 (3.56–6.61)	0.59
		30–300	15.24 (2.42)	−0.23 (0.89)	−1.21	0.24	0.1	0.93 (0.84–0.97)	0.96 (0.91–0.98)	0.19	4.18 (3.22–5.98)	0.53
		60–300	15.27 (2.63)	−0.26 (0.63)	−1.97	0.06	0.11	0.96 (0.91–0.99)	0.98 (0.95–0.99)	0.13	2.94 (2.26–4.2)	0.37
		90–150	15.28 (2.79)	−0.28 (1.1)	−1.17	0.26	0.11	0.91 (0.79–0.96)	0.95 (0.88–0.98)	0.24	5.16 (3.97–7.37)	0.65
		90–180	15.2 (2.65)	−0.19 (0.96)	−0.95	0.35	0.08	0.93 (0.83–0.97)	0.96 (0.91–0.98)	0.21	4.51 (3.47–6.45)	0.57
		90–210	15.17 (2.47)	−0.17 (1.00)	−0.78	0.45	0.07	0.92 (0.81–0.96)	0.936 (0.89–0.98)	0.21	4.7 (3.62–6.72)	0.59
		90–240	14.94 (2.52)	0.06 (0.79)	0.38	0.71	−0.03	0.95 (0.88–0.98)	0.97 (0.94–0.99)	0.17	3.74 (2.88–5.34)	0.47
		90–300	14.82 (2.57)	0.18 (0.74)	1.17	0.26	−0.07	0.95 (0.89–0.98)	0.98 (0.94–0.99)	0.16	3.51 (2.7–5.01)	0.44
		60–180	15.55 (2.69)	−0.54 (0.75)	−3.38	<0.01	0.22	0.94 (0.75–0.98)	0.97 (0.86–0.99)	0.16	3.49 (2.68–4.99)	0.45
P_max_ (Wkg^−2/3^)	12.72 (3.46)	60–240	12.77 (3.46)	−0.05 (1.01)	−0.23	0.82	0.01	0.96 (0.90–0.98)	0.98 (0.95–0.99)	2.01	5.62 (4.32–8.03)	0.37
		90–240	13.03 (3.59)	−0.31 (1.16)	−1.23	0.23	0.09	0.94 (0.87–0.98)	0.96 (0.83–0.99)	0.69	6.38 (4.91–9.12)	0.47
		60–180	11.96 (3.49)	0.76 (1.87)	1.91	0.07	−0.22	0.84 (0.65–0.93)	0.91 (0.79–0.96)	4.15	10.7 (8.23–15.28)	0.47

9p method, 9-point method; 2p method, 2-point method; COMB, a, combination of 2-point method; F_0_, maximal theoretical force; v_0_, maximal theoretical velocity; S_fv_, slope of force-velocity curve; P_max_, maximal theoretical power; ICCs, single intraclass correlation; ICCa, average intraclass correlation; t—t statistics; SEM, standard error of mean; ES, Cohen’s d coefficient; CV, coefficient of variation; SD, standard deviation; 95% CI, 95% confidence interval; MDC, minimal detectable change.

## 4 Discussion

In this study, we investigated the linearity of the F-v relationship, the concurrent validity of the simplified 2-point method for determining theoretical mechanical capacities in isokinetic single-joint tasks, and the discrepancies between KE and KF in the F-v profiles. The results showed: 1) high linearity of the 9-point method for KF and KE F-v relationship and 2) underestimation and moderate association between F_0_ and the isometric force; 3) significant differences between the KE and KF F-v parameters with a strong association between F_0_, v_0_ and P_max_; and 4) high concurrent validity of the 2-point method for KE and KF F-v parameters when using distant force and velocity points.

The presence of strong linearity of the F-v profile is preferred because it allows the use of the 2-point method and provides a reliable extrapolation of F_0_ and v_0_ (Jaric, 2015). So far, many studies have reported different curvatures of the relationship, such as hyperbolic and double hyperbolic, which makes the use of F-v profiles for evaluating the mechanical characteristics of single-joint tasks controversial. It is important to note that many studies have observed deviations from the linear relationship at high force and high velocity portions of the F-v profile (see Alcazar et al., 2019 for details). Bobbert (2012) described the F-v relationship single-joint segments in leg press as quasilinear (i.e., linear in the middle range and hyperbolic at the extreme ends). Similar results were observed in our study, as we found almost perfect linearity of KE (*r* = 0.97) and KF (*r* = 0.96) in the range between 30°/s and 300°/s, but underestimated the maximum F-capacity. One of the possible explanations could be that the nature of isokinetic movement enables detection of the Sfv, but not its actual maximal capacities (e.g., F_0_ and v_0_). This has already been observed in some studies evaluating multi-joint tasks, as Hahn et al. (2014) noted a hyperbolic F-v relationship at low velocity when performing leg press. Furthermore, Šarabon, Kozinc, and Marković (2020) found a significantly underestimated isometric force during the squat when comparing it to the F_0_ of a loaded countermovement jump. This linearity misconception of the F-v relationship across the entire velocity range may exist in both single- and multi-joint movements, but many studies evaluating bench press throw, KE, squat jump and countermovement jump at a certain range of velocity showed the relationship to be highly linear (R^2^ ∼ 0.95 and r ∼ 0.98) (Yamauchi et al., 2007; Sreckovic et al., 2015; Grbic et al., 2017b; García-Ramos et al., 2021; Piche et al., 2021). Although F_0_ and v_0_ may not directly reflect actual maximal force or velocity, there is a strong relationship between F-v parameters and neuromuscular capacity, as well as sport performance (Baena-Raya et al., 2021b). More importantly, by determining Sfv of KE and KF we can indicate if the muscle groups performing the movement are more “force” or “velocity” dominant. Based on the results, we can support our hypothesis that the F-v relationship of a single-joint at a limited range of velocities is not perfectly, but approximately linear. That observation supports the use of F-v profiling on single-joint tasks.

To our knowledge, Grbic et al. (2017a) were the first to evaluate F-v parameters of KE and KF and tested the concurrent validity of the 2-point method (60°/s and 180°/s) with respect to the four-point method. Later, Janicijevic et al. (2019) conducted a similar study using the same velocities (60°/s and 180°/s) in respect to the eight-point method, to evaluate its feasibility, sensitivity, generalizability, and linearity. In both studies, strong linearity of the F-v relationship and good validity of the respective 2-point method was observed, therefore the appeal for optimization of the 2-point method. Both studies observed satisfactory validity of the 2-point method, but only for the F_0_ and P_max_ in the study by Janicijevic et al. (2019). The main advantage of our study was that we observed high validity of 2-point methods for all KE FVP parameters when using distant loads. The combinations of the most distant velocities (30°/s, 60°/s, or 90°/s with a combination of 210°/s, 240°/s, or 300°/s) showed the highest agreement and the lowest within-individual and systematic error for the KE F_0_, v_0_, S_fv,_ and P_max_ and KF F_0_ and P_max_. In particular, 90°/s and 240°/s was the only combination that showed very high validity for measuring KE and also KF F_0_ and Pmax and could therefore be considered the most appropriate. The use of such distant F-v data was already supported on multi-joint tasks by Perez-Castilla et al. (2018) and García-Ramos et al. (2021) who observed that 0 and 75 kg for vertical jump and 20% and 70% 1RM for bench press provided the most valid results. Results from our study are a logical reflection of previous notions by Jaric (2016), who exposed that the error scores of the 2-point method had the smallest effect when the force and velocity data were obtained from two very reliable distant points. With this in mind, we can confirm our second hypothesis since the results of our study indicate that the 2-point method could be accurately applied in most cases when the low velocity is in the range between 30° and 90°/s and the second point is higher than 210°/s. However, these assumptions must be confirmed in future studies on larger samples and with different populations.

Compared to our results, Janicijevic et al. (2019) reported slightly larger F_0_ and similar v_0_ of KF and KE in both male and female participants. It could be intferred that their participants were better-trained individuals. Similar S_fv_ values observed between studies might indicate that the changes in the relationship between imposed velocity and hence force of KE and KF from the two studies are similar. For that reason isokinetic F-v profiling justifies its application in practice. Moreover, the simple 2-point method provides valid insight into the maximal theoretical mechanical capacity of KE and KF, which could be of great importance for the evaluation of muscular capacity. The significantly greater KE F_0_ in comparison to KF is consistent with other studies that have investigated the differences in isometric strength between quadriceps and hamstring muscles (Lord et al., 1992; Kong and Burns, 2010). It is evident that the F-v profile significantly differentiates between KE and KF, therefore our last hypothesis can be accepted. Interestingly, we have observed slightly (but non-significantly) larger KF v_0_ values. This was a reflection of hamstring ability of retaining a higher percentage of maximal external force production with increasing angular velocity during KF in comparison to KE. Therefore, KF S_fv_ was less steep than KE S_fv_, which suggests that the KF muscles might be more velocity dominant. Since the differences in v_0_ are relatively smaller than those in F_0_, KE possesses significantly higher P_max_ production capacity. The logical link between the F-v parameters, neuromuscular and anatomical characteristics of KE and KF muscles might be disrupted. Differences in relative force production capacity at a particular shortening velocity between KE and KF could be due to architectural differences between hamstring and quadriceps muscles and their different role with respect to overcoming gravity. Hamstrings are smaller in size, have smaller pennation angles and longer fiber lengths in comparison to quadriceps muscles (Kellis et al., 2012; Chleboun et al., 2014; E Lima et al., 2015; Maden-Wilkinson et al., 2021). Longer muscle fibers have more sarcomeres in series than shorter muscle fibers, meaning that at a given velocity sarcomeres shorten less for a given change in total muscle length in longer fibers. Therefore, hamstrings have a greater ability to sustain higher relative force with increasing velocity. Inversely, quadriceps have more potential to produce greater absolute F, since production capacity is proportional to muscle physiological cross section area:the larger the area, the greater the capacity to develop the external force. From that, it was suggested that KE are designed for producing large forces and KF for longer executions and faster movements (Lieber and Fridén, 2001). We infer that architectural characteristics of KE and KF are in a way reflected through F-v parameters, which jointly describe mechanical capacity at a wide range of velocity. For that purpose, it would be informative to use the F-v profile in future studies that aim to explore knee function. Specifically, the S_fv_ ratio between KE and KF could provide comprehensive insight into muscular strength (dis)balance, similar to a standard H/Q ratio. The main advantage of S_fv_ would be that it summarizes strength capacity across the total spectrum of measured concentric velocity, whereas the standard H/Q ratio usually considers strength capacity at one or two velocities (van Dyk et al., 2016). Moreover, practitioners could assess mechanical characteristics and function of the knee by using a simple, valid, fast, and non-fatigue prone 2-point F-v profiling method.

Despite the overall promising results of the study, several potential methodological limitations should be noted to properly interpret the data. Firstly, KE and KF strength was measured only in seated position, with hip flexed, and not also with hip extended. Predicted knee strength and F-v profles obtained might be therefore less sport specific. Secondly, the use of the linear method to interpret the F-v relationship between 30°/s and 300°/s seems to be appropriate since high linearity was observed at that range. However, it must be noted that F_0_ and v_0_ are theoretical values, and do not realistically reflect the true neuromuscular capacity of the muscles to produce maximal isometric force and maximal velocity, since the deviations from the linear F-v relationship are observed at its very ends. Thirdly, the data were obtained closer to F_0_, which could increase the bias of v_0_. Therefore, future studies might consider using even greater velocities when assessing the F-v profile of single-joint tasks. Next, it should be noted that the 2-point method was calculated from the data from a single 9-point method measurement, rather than from the data from a separate 2-point measurement, therefore fatigue might occur. Although this was not objectively measured, we believe that adequate rest between sets and randomized order of velocities negated the protocol-induced fatigue and potentially biased results. Finally, the participants were healthy, physically active young adults, so the high validity of the 2-point method can only be generalized to this population and may differ in more or less trained groups. Future studies should therefore investigate the validity and reliability of the method in athletes with different abilities.

Future studies should aim to evaluate a relationship and differences in the H/Q ratio and KF/KE ratio of the mechanical variables of the F-v profile and to establish reference values (e.g., taking into account the effects of training status, maturity, gender, sport type, injury, etc.) for the F-v profile (F0, v0, Sfv, and Pmax) of KF and KE to allow reliable classification of the specific muscle group as force or velocity dominant. Conclusions regarding optimal training modes for these muscle groups could then be drawn and tested. The next important aspect of research could be the relationship between the structural and neuromuscular properties and the F-v profile of KF and KE.

## 5 Conclusion

In conclusion, this study confirms a high linearity of the F-v relationship in KE and KF tasks and a high concurrent validity of the 2-point method for evaluating the F-v profile of single-joint tasks when distant force and velocity points are used. It is difficult to determine the most optimal combination of isokinetic velocities for the test procedure, as different combinations yielded valid results for different F-v parameters. However, we can recommend using combinations of velocities below 90°/s (ideally 30°/s) and above 210°/s when possible. The 2-point method for evaluating the F-v profile of a single joint is faster, simpler, less fatiguing, and a good alternative to the multi-point method when dealing with single joint tasks. For this reason, practitioners should consider it when measuring the mechanical capacity of muscles. In addition, the F-v profile, particularly the S_fv_, has the potential to differentiate between the mechanical characteristics of KE and KF muscles and should therefore be further evaluated as a performance descriptor in studies investigating knee function and injury prevention*.*

## Practical Application

Two important practical conclusions can be drawn from the results of this study, which complement each other. The F-v profile of the isokinetic KE and KF jointly describe the mechanical capacity of the knee extensors and knee flexors over a wide range of velocities and could therefore be used to study knee function in terms of performance or injury prevention. And for this purpose, practitioners can use a highly valid 2-point protocol consisting of two widely distant loads rather than a time-consuming and fatigue-prone multi-point protocol.

## Data Availability

The raw data supporting the conclusions of this article will be made available by the authors, without undue reservation.

## References

[B1] Alcazar J. Csapo R. Ara I. Alegre L. M. (2019). On the Shape of the Force-Velocity Relationship in Skeletal Muscles: The Linear, the Hyperbolic, and the Double-Hyperbolic. Front. Physiol. 10, 1–21. 10.3389/fphys.2019.00769 31275173 PMC6593051

[B2] Baena-raya A. García-Mateo P. García-Ramos A. Rodríguez-Pérez M. A. Soriano-maldonado A. (2021a). Delineating the Potential of the Vertical and Horizontal Force-Velocity Profile for Optimizing Sport Performance: A Systematic Review. J. Sports Sci. 40, 331–344. 10.1080/02640414.2021.1993641 34727836

[B3] Baena-Raya A. Rodríguez-Pérez M. A. Jiménez-Reyes P. Soriano-Maldonado A. (2021b). Maximizing Acceleration and Change of Direction in Sport: A Case Series to Illustrate How the Force-Velocity Profile Provides Additional Information to that Derived from Linear Sprint Time. Ijerph 18, 6140. 10.3390/ijerph18116140 34200129 PMC8201263

[B4] Baroni B. M. Ruas C. V. Ribeiro-Alvares J. B. Pinto R. S. (2020). Hamstring-to-quadriceps Torque Ratios of Professional Male Soccer Players: A Systematic Review. J. strength Cond. Res. 34, 281–293. 10.1519/JSC.0000000000002609 29794893

[B5] Bobbert M. F. (2012). Why Is the Force-Velocity Relationship in Leg Press Tasks Quasi-Linear rather Than Hyperbolic? J. Appl. Physiology 112, 1975–1983. 10.1152/japplphysiol.00787.2011 22442026

[B6] Chleboun G. S. France A. R. Crill M. T. Braddock H. K. Howell J. N. (2001). *In Vivo* Measurement of Fascicle Length and Pennation Angle of the Human Biceps Femoris Muscle. Cells Tissues Organs 169, 401–409. 10.1159/000047908 11490120

[B7] Cohen J. (1988). Statistical Power Analysis for the Behavioral Sciences. 2nd ed.. New York: Lawrence Erlbaum Associates.

[B8] E Lima K. M. M. Carneiro S. P. de S. Alves D. Peixinho C. C. De Oliveira L. F. (2015). Assessment of Muscle Architecture of the Biceps Femoris and Vastus Lateralis by Ultrasound after a Chronic Stretching Program. Clin. J. Sport Med. 25, 55–60. 10.1097/JSM.0000000000000069 24451696

[B9] Finni T. Ikegawa S. Lepola V. Komi P. V. (2003). Comparison of Force-Velocity Relationships of Vastus Lateralis Muscle in Isokinetic and in Stretch-Shortening Cycle Exercises. Acta Physiol. Scand. 177, 483–491. 10.1046/j.1365-201X.2003.01069.x 12648166

[B10] García-Ramos A. Jaric S. Padial P. Feriche B. (2016). Force-velocity Relationship of Upper Body Muscles: Traditional versus Ballistic Bench Press. J. Appl. Biomech. 32, 178–185. 10.1123/jab.2015-0162 26540734

[B11] Garcia-Ramos A. Jaric S. (2018). Two-point Method: A Quick and Fatigue-free Procedure for Assessment of Muscle Mechanical Capacities and the 1 Repetition Maximum. Strength Cond. J. 40, 54–66. 10.1519/ssc.0000000000000359

[B12] García-Ramos A. Pérez-Castilla A. Jaric S. (2021). Optimisation of Applied Loads when Using the Two-point Method for Assessing the Force-Velocity Relationship during Vertical Jumps. Sports Biomech. 20, 274–289. 10.1080/14763141.2018.1545044 30540216

[B13] Grbic V. Djuric S. Knezevic O. Mirkov D. Nedeljkovic A. Jaric S. (2017a). A Novel Two-Velocity Method for Elaborate Isokinetic Testing of Knee Extensors. Int. J. Sports Med. 38, 741–746. 10.1055/s-0043-113043 28768340

[B14] Grbic V. Djuric S. Knezevic O. Mirkov D. Nedeljkovic A. Jaric S. (2017b). A Novel Two-Velocity Method for Elaborate Isokinetic Testing of Knee Extensors. Int. J. Sports Med. 38, 741–746. 10.1055/s-0043-113043 28768340

[B15] Hahn D. Herzog W. Schwirtz A. (2014). Interdependence of Torque, Joint Angle, Angular Velocity and Muscle Action during Human Multi-Joint Leg Extension. Eur. J. Appl. Physiol. 114, 1691–1702. 10.1007/s00421-014-2899-5 24819448

[B16] Hald R. D. Bottjen E. J. (1987). Effect of Visual Feedback on Maximal and Submaximal Isokinetic Test Measurements of Normal Quadriceps and Hamstrings. J. Orthop. Sports Phys. Ther. 9, 86–93. 10.2519/jospt.1987.9.2.86 18797018

[B17] Harris R. T. Dudley G. A. (1994). Factors Limiting Force during Slow, Shortening Actions of the Quadriceps Femoris Muscle Group *in Vivo* . Acta Physiol. Scand. 152, 63–71. 10.1111/j.1748-1716.1994.tb09785.x 7810333

[B18] Haugen T. A. Breitschädel F. Seiler S. (2019). Sprint Mechanical Variables in Elite Athletes: Are Force-Velocity Profiles Sport Specific or Individual? PLoS One 14, e0215551. 10.18710/PJONBM10.1371/journal.pone.0215551 31339890 PMC6655540

[B19] Hill A. V. (1938). The Heat of Shortening and the Dynamic Constants of Muscle. Proc. R. Soc. Lond. B 126, 136–195. 10.1098/rspb.1938.0050

[B20] Hopkins W. G. Marshall S. W. Batterham A. M. Hanin J. (2009). Progressive Statistics for Studies in Sports Medicine and Exercise Science. Med. Sci. Sports Exerc. 41, 3–12. 10.1249/MSS.0b013e31818cb278 19092709

[B21] Janicijevic D. García-Ramos A. Knezevic O. M. Mirkov D. M. (2019). Feasibility of the Two-point Method for Assessing the Force-Velocity Relationship during Lower-Body and Upper-Body Isokinetic Tests. J. Sports Sci. 37, 2396–2402. 10.1080/02640414.2019.1636523 31256708

[B22] Janicijevic D. Knezevic O. M. Mirkov D. M. Pérez-Castilla A. Petrovic M. Samozino P. (2020). Assessment of the Force-Velocity Relationship during Vertical Jumps: Influence of the Starting Position, Analysis Procedures and Number of Loads. Eur. J. Sport Sci. 20, 614–623. 10.1080/17461391.2019.1645886 31314671

[B23] Jaric S. (2015). Force-velocity Relationship of Muscles Performing Multi-Joint Maximum Performance Tasks. Int. J. Sports Med. 36, 699–704. 10.1055/s-0035-1547283 25806588

[B24] Jaric S. (2002). Muscle Strength Testing. Sports Med. 32, 615–631. 10.2165/00007256-200232100-00002 12141882

[B25] Jaric S. (2016). Two-Load Method for Distinguishing Between Muscle Force, Velocity, and Power-Producing Capacities. Sports Med. 46, 1585–1589. 10.1007/s40279-016-0531-z.Two-loads 27075326 PMC5056118

[B26] Jiménez-Reyes P. Samozino P. Pareja-Blanco F. Conceição F. Cuadrado-Peñafiel V. Gonzalez-Badillo J. J. (2016). Validity of a Simple Method for Measuring Force-Velocity-Power Profile in Countermovement Jump. Int. J. Sports Physiol. Perform. 14, 156–162. Available at: https://www.cochranelibrary.com/central/doi/10.1002/central/CN-01787161/full . 10.1123/ijspp.2015-0484 27002490

[B27] Kellis E. Galanis N. Kapetanos G. Natsis K. (2012). Architectural Differences between the Hamstring Muscles. J. Electromyogr. Kinesiol. 22, 520–526. 10.1016/j.jelekin.2012.03.012 22564790

[B28] Kellis E. Galanis N. Kofotolis N. (2019). Hamstring-to-quadriceps Ratio in Female Athletes with a Previous Hamstring Injury, Anterior Cruciate Ligament Reconstruction, and Controls. Sports 7, 214–310. 10.3390/sports7100214 PMC6835705 31569442

[B29] Kong P. W. Burns S. F. (2010). Bilateral Difference in Hamstrings to Quadriceps Ratio in Healthy Males and Females. Phys. Ther. Sport 11, 12–17. 10.1016/j.ptsp.2009.09.004 20129118

[B30] Koo T. K. Li M. Y. (2016). A Guideline of Selecting and Reporting Intraclass Correlation Coefficients for Reliability Research. J. Chiropr. Med. 15, 155–163. 10.1016/j.jcm.2016.02.012 27330520 PMC4913118

[B31] Lee J. W. Y. Mok K.-M. Chan H. C. K. Yung P. S. H. Chan K.-M. (2018). Eccentric Hamstring Strength Deficit and Poor Hamstring-To-Quadriceps Ratio Are Risk Factors for Hamstring Strain Injury in Football: A Prospective Study of 146 Professional Players. J. Sci. Med. Sport 21, 789–793. 10.1016/j.jsams.2017.11.017 29233665

[B32] Lemaire A. Ripamonti M. Ritz M. Rahmani A. (2014). Agreement of Three vs. Eight Isokinetic Preset Velocities to Determine Knee Extensor Torque- and Power-Velocity Relationships. Ies 22, 1–7. 10.3233/IES-130524

[B33] Lieber R. L. Fridén J. (2001). Clinical Significance of Skeletal Muscle Architecture. Clin. Orthop. Relat. Res. 383, 140–151. 10.1097/00003086-200102000-00016 11210948

[B34] Lindberg K. Solberg P. Rønnestad B. R. Frank M. T. Larsen T. Abusdal G. (2021). Should We Individualize Training Based on Force‐velocity Profiling to Improve Physical Performance in Athletes? Scand. J. Med. Sci. Sports 31, 2198–2210. 10.1111/sms.14044 34473848

[B35] López-Segovia M. Marques M. Van Den Tillaar R. González-Badillo J. (2011). Relationships between Vertical Jump and Full Squat Power Outputs with Sprint Times in U21 Soccer Players. J. Hum. Kinet. 30, 135–144. 10.2478/v10078-011-0081-2 23487438 PMC3588648

[B36] Lord J. P. Aitkens S. G. McCrory M. A. Bernauer E. M. (1992). Isometric and Isokinetic Measurement of Hamstring and Quadriceps Strength. Archives Phys. Med. Rehabilitation 73, 324–330. 10.1016/0003-9993(92)90004-G 1554304

[B37] Maden-Wilkinson T. M. Balshaw T. G. Massey G. J. Folland J. P. (2021). Muscle Architecture and Morphology as Determinants of Explosive Strength. Eur. J. Appl. Physiol. 121, 1099–1110. 10.1007/s00421-020-04585-1 33458800 PMC7966212

[B38] Morales-Artacho A. Ramos A. Pérez-Castilla A. Padial P. Argüelles-Cienfuegos J. De La Fuente B. (2018). Associations of the Force-Velocity Profile with Isometric Strength and Neuromuscular Factors. Int. J. Sports Med. 39, 984–994. 10.1055/a-0644-3742 30290374

[B39] Myer G. D. Ford K. R. Barber Foss K. D. Liu C. Nick T. G. Hewett T. E. (2009). The Relationship of Hamstrings and Quadriceps Strength to Anterior Cruciate Ligament Injury in Female Athletes. Clin. J. Sport Med. 19, 3–8. 10.1097/JSM.0b013e318190bddb 19124976 PMC9928500

[B40] Pérez-Castilla A. Jaric S. Feriche B. Padial P. García-Ramos A. (2018). Evaluation of Muscle Mechanical Capacities through the Two-Load Method: Optimization of the Load Selection. J. Strength Cond. Res. 32, 1245–1253. 10.1519/JSC.0000000000001969 28475551

[B41] Piche E. Chorin F. Gerus P. Jaafar A. Reneaud N. Guerin O. (2021). Validity of a Simple Sit-To-Stand Method for Assessing Force-Velocity Profile in Older Adults. Exp. Gerontol. 156, 111595. 10.1016/j.exger.2021.111595 34673170

[B42] Samozino P. Morin J.-B. Hintzy F. Belli A. (2008). A Simple Method for Measuring Force, Velocity and Power Output during Squat Jump. J. Biomechanics 41, 2940–2945. 10.1016/j.jbiomech.2008.07.028 18789803

[B43] Samozino P. Rejc E. Di Prampero P. E. Belli A. Morin J.-B. (2012). Optimal Force-Velocity Profile in Ballistic Movements-Altius. Med. Sci. Sports Exerc. 44, 313–322. 10.1249/MSS.0b013e31822d757a 21775909

[B44] Šarabon N. Kozinc Ž. Marković G. (2020). Force-velocity Profile during Vertical Jump Cannot Be Assessed Using Only Bodyweight Jump and Isometric Maximal Voluntary Contraction Tasks. Sci. Rep. 10, 1–12. 10.1038/s41598-020-76262-4 33154481 PMC7645790

[B45] Seger J. Y. Thorstensson A. (2000). Electrically Evoked Eccentric and Concentric Torque-Velocity Relationships in Human Knee Extensor Muscles. Acta Physiol. Scand. 169, 63–69. 10.1046/j.1365-201X.2000.00694.x 10759612

[B46] Sreckovic S. Cuk I. Djuric S. Nedeljkovic A. Mirkov D. Jaric S. (2015). Evaluation of Force-Velocity and Power-Velocity Relationship of Arm Muscles. Eur. J. Appl. Physiol. 115, 1779–1787. 10.1007/s00421-015-3165-1 25828144

[B47] van Dyk N. Bahr R. Whiteley R. Tol J. L. Kumar B. D. Hamilton B. (2016). Hamstring and Quadriceps Isokinetic Strength Deficits are Weak Risk Factors for Hamstring Strain Injuries. Am. J. Sports Med. 44, 1789–1795. 10.1177/0363546516632526 27002102

[B48] Wickiewicz T. L. Roy R. R. Powell P. L. Perrine J. J. Edgerton V. R. (1984). Muscle Architecture and Force-Velocity Relationships in Humans. J. Appl. Physiology 57, 435–443. 10.1152/jappl.1984.57.2.435 6469814

[B49] Yamauchi J. Mishima C. Fujiwara M. Nakayama S. Ishii N. (2007). Steady-state Force-Velocity Relation in Human Multi-Joint Movement Determined with Force Clamp Analysis. J. Biomechanics 40, 1433–1442. 10.1016/j.jbiomech.2006.06.010 16989841

