# Supplementary material for: The Validity of the 2-Point Method for Assessing the Force-Velocity Relationship of the Knee Flexors and Knee Extensors: The Relevance of Distant Force-Velocity Testing
Source: Front Physiol. 2022 Jun 24;13:849275. doi: 10.3389/fphys.2022.849275 (PMC9263277; doi:10.3389/fphys.2022.849275)
Supplement: Supplementary file 1 [file Table1.DOCX]

**Supplementary Material**

| **9-point method** | | **2-point methods** | |  | **Systematic error** | | | |  | **Intra-class correlation** | |  | **Within individual error** | |
| --- | --- | --- | --- | --- | --- | --- | --- | --- | --- | --- | --- | --- | --- | --- |
|  |  | **Combination** | **Mean (±SD)** |  | **Mean bias (±SD)** | **t** | **sig** | **ES** |  | **ICCs (95% CI)** | **ICCa (95% CI)** |  | **SEM** | **CV % (95% CI)** |
| **F_0_ (Nkg^-2/3^)** | **27.98 (3.28)** | 30-60 | 28.31 (3.51) |  | -0.33 (2.98) | -0.515 | 0.612 | 0.10 |  | 0.62 (0.28-0.82) | 0.77 (0.43-0.9) |  | 0.64 | 7.5 (5.77-10.72) |
|  |  | 30-90 | 28.76 (3.41) |  | -0.78 (2.31) | -1.59 | 0.13 | 0.23 |  | 0.75 (0.49-0.89) | 0.86 (0.66-0.94) |  | 0.49 | 5.75 (4.42-8.21) |
|  |  | 30-120 | 28.79 (3.2) |  | -0.81 (1.92) | -1.98 | 0.06 | 0.25 |  | 0.81 (0.58-0.92) | 0.89 (0.73-0.96) |  | 0.41 | 4.78 (3.68-6.83) |
|  |  | 30-150 | 28.82 (3.14) |  | -0.84 (1.68) | -2.340 | 0.029 | 0.26 |  | 0.84 (0.62-0.93) | 0.91 (0.77-0.97) |  | 0.36 | 4.18 (3.21-5.97) |
|  |  | 30-180 | 28.71 (3.12) |  | -0.73 (1.58) | -2.164 | 0.042 | 0.23 |  | 0.86 (0.67-0.94) | 0.93 (0.8-0.97) |  | 0.34 | 3.94 (3.03-5.63) |
|  |  | 30-210 | 28.62 (3.16) |  | -0.64 (1.5) | -2.00 | 0.06 | 0.20 |  | 0.88 (0.72-0.95) | 0.94 (0.84-0.97) |  | 0.32 | 3.75 (2.89-5.36) |
|  |  | 30-240 | 28.58 (3.12) |  | -0.6 (1.45) | -1.95 | 0.06 | 0.19 |  | 0.89 (0.74-0.95) | 0.94 (0.85-0.98) |  | 0.31 | 3.61 (2.78-5.16) |
|  |  | 60-90 | 30.13 (4.7) |  | -2.15 (2.66) | -3.787 | 0.001 | 0.53 |  | 0.69 (0.2-0.88) | 0.82 (0.34-0.94) |  | 0.57 | 6.47 (4.98-9.25) |
|  |  | 60-120 | 29.75 (4.12) |  | -1.77 (2.01) | -4.121 | 0.000 | 0.48 |  | 0.77 (0.25-0.92) | 0.87 (0.41-0.96) |  | 0.43 | 4.94 (3.8-7.05) |
|  |  | 60-150 | 29.66 (3.86) |  | -1.68 (1.31) | -6.043 | 0.000 | 0.47 |  | 0.84 (0.09-0.96) | 0.92 (0.17-0.98) |  | 0.28 | 3.2 (2.47-4.58) |
|  |  | 60-210 | 29.06 (3.89) |  | -1.08 (1.12) | -4.519 | 0.000 | 0.30 |  | 0.91 (0.51-0.97) | 0.95 (0.67-0.99) |  | 0.24 | 2.47 (1.9-3.53) |
|  |  | 60-240 | 28.95 (3.79) |  | -0.97 (1) | -4.559 | 0.000 | 0.27 |  | 0.93 (0.56-0.98) | 0.96 (0.72-0.99) |  | 0.21 | 2.31 (1.78-3.3) |
|  |  | 60-300 | 28.58 (3.7) |  | -0.6 (0.92) | -3.052 | 0.006 | 0.17 |  | 0.95 (0.84-0.98) | 0.98 (0.91-0.99) |  | 0.20 | 9.93 (7.64-14.19) |
|  |  | 90-120 | 29 (4.97) |  | -1.02 (4) | -1.193 | 0.246 | 0.24 |  | 0.54 (0.18-0.78) | 0.70 (0.3-0.88) |  | 0.85 | 6.53 (5.02-9.33) |
|  |  | 90-150 | 29.09 (4.23) |  | -1.11 (2.63) | -1.972 | 0.062 | 0.29 |  | 0.73 (0.45-0.88) | 0.85 (0.63-0.94) |  | 0.56 | 5.13 (3.95-7.33) |
|  |  | 90-180 | 28.49 (3.88) |  | -0.51 (2.05) | -1.18 | 0.25 | 0.14 |  | 0.84 (0.65-0.93) | 0.91 (0.79-0.96) |  | 0.44 | 5.13 (3.95-7.33) |
|  |  | 120-150 | 29.23 (7.04) |  | -1.25 (5.9) | -0.994 | 0.331 | 0.23 |  | 0.42 (0.02-0.71) | 0.59 (0.03-0.83) |  | 1.26 | 14.58 (11.22-20.84) |
|  |  | 120-180 | 27.99 (5) |  | -0.01 (3.44) | -0.014 | 0.989 | 0.00 |  | 0.68 (0.36-0.85) | 0.81 (0.53-0.92) |  | 0.73 | 8.69 (6.68-12.42) |
|  |  | 120-210 | 27.45 (4.66) |  | 0.53 (2.39) | 1.04 | 0.31 | -0.13 |  | 0.82 (0.63-0.92) | 0.9 (0.77-0.96) |  | 0.51 | 6.09 (4.68-8.7) |
|  |  | 120-240 | 27.34 (4.39) |  | 0.64 (2.23) | 1.35 | 0.19 | -0.16 |  | 0.83 (0.64-0.93) | 0.91 (0.78-0.96) |  | 0.47 | 5.69 (4.38-8.13) |
|  |  | 120-300 | 26.63 (4.04) |  | 1.35 (1.89) | 3.347 | 0.003 | -0.37 |  | 0.82 (0.46-0.93) | 0.9 (0.63-0.96) |  | 0.40 | 4.88 (3.76-6.98) |
|  |  | 150-180 | 26.13 (4.91) |  | 1.85 (3.66) | 2.373 | 0.027 | -0.44 |  | 0.57 (0.2-0.8) | 0.73 (0.34-0.89) |  | 0.78 | 9.56 (7.35-13.66) |
|  |  | 150-210 | 25.89 (4.92) |  | 2.08 (2.66) | 3.679 | 0.001 | -0.50 |  | 0.72 (0.24-0.89) | 0.83 (0.39-0.94) |  | 0.57 | 6.97 (5.37-9.97) |
|  |  | 150-240 | 26.08 (4.34) |  | 1.9 (2.18) | 4.082 | 0.001 | -0.49 |  | 0.75 (0.23-0.91) | 0.86 (0.38-0.95) |  | 0.47 | 5.71 (4.39-8.15) |
|  |  | 150-300 | 25.33 (3.86) |  | 2.64 (1.77) | 7.015 | 0.000 | -0.74 |  | 0.69 (-0.07-0.91) | 0.82 (-0.15-0.95) |  | 0.38 | 4.69 (3.61-6.7) |
|  |  | 180-210 | 25.56 (7.66) |  | 2.41 (5.71) | 1.984 | 0.061 | -0.41 |  | 0.5 (0.13-0.75) | 0.67 (0.23-0.86) |  | 1.22 | 15.08 (11.6-21.55) |
|  |  | 180-240 | 26.04 (5.15) |  | 1.94 (3.13) | 2.904 | 0.008 | -0.45 |  | 0.68 (0.3-0.86) | 0.81 (0.46-0.93) |  | 0.67 | 8.19 (6.3-11.7) |
|  |  | 180-300 | 24.94 (4.06) |  | 3.04 (2.04) | 6.974 | 0.000 | -0.82 |  | 0.64 (-0.08-0.89) | 0.78 (-0.18-0.94) |  | 0.44 | 5.46 (4.2-7.81) |
|  |  | 210-240 | 26.68 (5.18) |  | 1.3 (4.73) | 1.291 | 0.211 | -0.30 |  | 0.4 (0-0.69) | 0.57 (0-0.82) |  | 1.01 | 12.23 (9.41-17.47) |
|  |  | 210-300 | 24.59 (3.29) |  | 3.39 (2.46) | 6.452 | 0.000 | -1.03 |  | 0.47 (-0.1-0.79) | 0.64 (-0.23-0.89) |  | 0.53 | 6.63 (5.1-9.48) |
|  |  | 240-300 | 23.1 (3.54) |  | 4.88 (2.76) | 8.287 | 0.000 | -1.43 |  | 0.34 (-0.1-0.71) | 0.5 (-0.22-0.83) |  | 0.59 | 7.65 (5.89-10.93) |

**Table 1.** Excluded KE F_0_ 2-point methods.

| **9-point method** | | **2-point methods** | |  | **Systematic error** | | | |  | **Intra-class correlation** | |  | **Within individual error** | |
| --- | --- | --- | --- | --- | --- | --- | --- | --- | --- | --- | --- | --- | --- | --- |
|  |  | **Combination** | **Mean (±SD)** |  | **Mean bias (±SD)** | **t** | **sig** | **ES** |  | **ICCs (95% CI)** | **ICCa (95% CI)** |  | **SEM** | **CV % (95% CI)** |
| **v_0_ (ms^-1^)** | **3.16 (0.53)** | 30-60 | 12.21 (54.32) |  | -9.05 (54.29) | -0.78 | 0.44 | 0.24 |  | 0.00 (-0.42-0.42) | 0.00 (-1.46-0.59) |  | 11.57 | 499.41 (384.22-713.69) |
|  |  | 30-90 | 3.79 (5.5) |  | -0.63 (5.34) | -0.55 | 0.59 | 0.16 |  | 0.07 (-0.37-0.48) | 0.13 (-1.18-0.64) |  | 1.14 | 108.61 (83.56-155.21) |
|  |  | 30-120 | 4.35 (5.66) |  | -1.19 (5.42) | -1.03 | 0.31 | 0.30 |  | 0.09 (-0.33-0.49) | 0.17 (-0.98-0.65) |  | 1.15 | 101.96 (78.45-145.71) |
|  |  | 30-150 | 2.87 (0.95) |  | 0.29 (0.53) | 2.51 | 0.02 | -0.37 |  | 0.72 (0.39-0.88) | 0.84 (0.56-0.93) |  | 0.11 | 12.5 (9.61-17.86) |
|  |  | 30-180 | 2.96 (0.83) |  | 0.2 (0.45) | 2.06 | 0.05 | -0.28 |  | 0.77 (0.5-0.9) | 0.87 (0.67-0.95) |  | 0.10 | 10.43 (8.02-14.9) |
|  |  | 30-210 | 3.07 (0.74) |  | 0.09 (0.31) | 1.43 | 0.17 | -0.15 |  | 0.88 (0.73-0.95) | 0.94 (0.85-0.97) |  | 0.07 | 7.03 (5.41-10.05) |
|  |  | 30-300 | 3.39 (0.66) |  | -0.21 (0.19) | -5.02 | 0.00 | 0.39 |  | 0.9 (0.33-0.97) | 0.95 (0.5-0.99) |  | 0.04 | 4.03 (3.06-5.88) |
|  |  | 60-90 | -4.4 (24.12) |  | 7.56 (24.18) | 1.47 | 0.16 | -0.44 |  | 0.00 (-0.39-0.4) | -0.01 (-1.27-0.57) |  | 5.15 | 2759.27 (2122.85-3943.18) |
|  |  | 60-120 | 2.87 (1.35) |  | 0.29 (1.25) | 1.10 | 0.28 | -0.28 |  | 0.26 (-0.16-0.61) | 0.42 (-0.38-0.76) |  | 0.27 | 29.19 (22.46-41.71) |
|  |  | 60-150 | 2.61 (0.62) |  | 0.55 (0.38) | 6.72 | 0.00 | -0.95 |  | 0.54 (-0.1-0.84) | 0.7 (-0.21-0.91) |  | 0.08 | 9.41 (7.24-13.44) |
|  |  | 60-210 | 2.95 (0.5) |  | 0.21 (0.27) | 3.75 | 0.00 | -0.41 |  | 0.8 (0.37-0.93) | 0.89 (0.54-0.96) |  | 0.06 | 6.21 (4.78-8.88) |
|  |  | 60-240 | 3 (0.46) |  | 0.16 (0.2) | 3.63 | 0.00 | -0.31 |  | 0.88 (0.55-0.96) | 0.94 (0.71-0.98) |  | 0.04 | 4.64 (3.57-6.63) |
|  |  | 60-300 | 3.3 (0.47) |  | -0.14 (0.14) | -4.82 | 0.00 | 0.28 |  | 0.93 (0.5-0.98) | 0.96 (0.67-0.99) |  | 0.03 | 3.04 (2.34-4.35) |
|  |  | 90-120 | 3.42 (4.94) |  | -0.26 (4.83) | -0.26 | 0.80 | 0.08 |  | 0.06 (-0.39-0.47) | 0.11 (-1.28-0.64) |  | 1.03 | 103.78 (79.85-148.31) |
|  |  | 90-150 | 2.85 (0.89) |  | 0.31 (0.7) | 2.08 | 0.05 | 12.70 |  | 0.51 (0.14-0.76) | 0.67 (0.24-0.86) |  | 0.15 | 16.5 (12.7-23.58) |
|  |  | 90-180 | 2.97 (0.74) |  | 0.19 (0.58) | 1.50 | 0.15 | -0.29 |  | 0.59 (0.24-0.8) | 0.74 (0.39-0.89) |  | 0.12 | 13.28 (10.22-18.98) |
|  |  | 90-210 | 3.1 (0.55) |  | 0.06 (0.35) | 0.82 | 0.42 | -0.11 |  | 0.79 (0.56-0.91) | 0.88 (0.72-0.95) |  | 0.08 | 7.99 (6.15-11.42) |
|  |  | 90-240 | 3.14 (0.51) |  | 0.02 (0.3) | 0.38 | 0.70 | -0.04 |  | 0.85 (0.66-0.93) | 0.92 (0.8-0.97) |  | 0.06 | 6.61 (5.09-9.45) |
|  |  | 90-300 | 3.42 (0.5) |  | -0.26 (0.21) | -5.99 | 0.00 | 0.51 |  | 0.82 (0.06-0.95) | 0.90 (0.12-0.97) |  | 0.04 | 4.45 (3.42-6.35) |
|  |  | 120-150 | 2 (4.13) |  | 1.16 (4.26) | 1.28 | 0.22 | -0.39 |  | -0.05 (-0.44-0.37) | -0.1 (-1.55-0.54) |  | 0.91 | 116.84 (89.89-166.97) |
|  |  | 120-180 | -4.08 (33.41) |  | 7.24 (33.53) | 1.01 | 0.32 | -0.31 |  | -0.01 (-0.42-0.41) | -0.01 (-1.44-0.58) |  | 7.15 | 5164.23 (3973.11-7380.01) |
|  |  | 120-210 | 3.27 (0.74) |  | -0.11 (0.51) | -1.05 | 0.30 | 0.18 |  | 0.69 (0.40-0.86) | 0.82 (0.57-0.92) |  | 0.11 | 11.19 (8.61-15.99) |
|  |  | 120-240 | 3.27 (0.66) |  | -0.11 (0.42) | -1.19 | 0.25 | 0.18 |  | 0.75 (0.49-0.88) | 0.85 (0.65-0.94) |  | 0.09 | 9.34 (7.18-13.34) |
|  |  | 120-300 | 3.55 (0.61) |  | -0.39 (0.33) | -5.60 | 0.00 | 0.69 |  | 0.68 (-0.02-0.89) | 0.81 (-0.03-0.94) |  | 0.07 | 6.99 (5.38-10) |
|  |  | 150-180 | 6.31 (12.24) |  | -3.15 (12.26) | -1.21 | 0.24 | 0.36 |  | 0.00 (-0.40-0.41) | 0.00 (-1.34-0.58) |  | 2.61 | 183.03 (140.82-261.57) |
|  |  | 150-210 | 3.8 (1.19) |  | -0.64 (1.16) | -2.59 | 0.02 | 0.70 |  | 0.17 (-0.17-0.51) | 0.29 (-0.41-0.68) |  | 0.25 | 23.61 (18.17-33.75) |
|  |  | 150-240 | 3.47 (0.56) |  | -0.31 (0.44) | -3.25 | 0.00 | 0.56 |  | 0.59 (0.15-0.82) | 0.74 (0.27-0.9) |  | 0.09 | 9.43 (7.25-13.47) |
|  |  | 150-300 | 3.73 (0.53) |  | -0.57 (0.31) | -8.51 | 0.00 | 1.06 |  | 0.53 (-0.09-0.84) | 0.69 (-0.21-0.92) |  | 0.07 | 6.44 (4.95-9.2) |
|  |  | 180-210 | 0.6 (16.71) |  | 2.56 (16.78) | 0.72 | 0.48 | -0.22 |  | -0.01 (-0.43-0.41) | -0.02 (-1.53-0.58) |  | 3.58 | 631.52 (485.86-902.49) |
|  |  | 180-240 | 3.71 (1.37) |  | -0.55 (1.2) | -2.15 | 0.04 | 0.53 |  | 0.3 (-0.07-0.62) | 0.47 (-0.16-0.77) |  | 0.26 | 24.7 (19-35.3) |
|  |  | 180-300 | 3.83 (0.67) |  | -0.67 (0.43) | -7.30 | 0.00 | 1.11 |  | 0.47 (-0.1-0.8) | 0.64 (-0.23-0.89) |  | 0.09 | 8.77 (6.75-12.54) |
|  |  | 210-240 | 3.89 (1.95) |  | -0.73 (2.04) | -1.67 | 0.11 | 0.51 |  | -0.01 (-0.38-0.38) | -0.03 (-1.24-0.55) |  | 0.43 | 40.92 (31.48-58.48) |
|  |  | 210-300 | 3.88 (0.62) |  | -0.72 (0.43) | -7.79 | 0.00 | 1.25 |  | 0.41 (-0.1-0.76) | 0.58 (-0.23-0.86) |  | 0.09 | 8.73 (6.71-12.47) |
|  |  | 240-300 | 4.27 (0.86) |  | -1.11 (0.62) | -8.34 | 0.00 | 1.54 |  | 0.29 (-0.1-0.66) | 0.44 (-0.22-0.79) |  | 0.13 | 11.88 (9.14-16.98) |

**Table 2**. Excluded KE v_0_ 2-point methods.

| **9-point method** | | **2-point methods** | |  | **Systematic error** | | | |  | **Intra-class correlation** | |  | **Within individual error** | |
| --- | --- | --- | --- | --- | --- | --- | --- | --- | --- | --- | --- | --- | --- | --- |
|  |  | **Combination** | **Mean (±SD)** |  | **Mean bias (±SD)** | **t** | **sig** | **ES** |  | **ICCs (95% CI)** | **ICCa (95% CI)** |  | **SEM** | **CV % (95% CI)** |
| **S_fv_ (Nms^-1^kg^-2/3^)** | **-9.06 (1.66)** | 30-60 | -8.18 (10.37) |  | -0.87 (2.13) | -0.41 | 0.68 | 0.12 |  | 0.10 (-0.34-0.5) | 0.19 (-1.05-0.67) |  | 2.13 | 81.81 (62.94-116.91) |
|  |  | 30-90 | -10.7 (6.53) |  | 1.64 (1.26) | 1.31 | 0.21 | -0.35 |  | 0.23 (-0.19-0.58) | 0.37 (-0.46-0.73) |  | 1.26 | 42.25 (32.51-60.38) |
|  |  | 30-120 | -10.83 (4.54) |  | 1.78 (0.8) | 2.23 | 0.04 | -0.52 |  | 0.36 (-0.02-0.66) | 0.53 (-0.04-0.8) |  | 0.80 | 26.61 (20.48-38.03) |
|  |  | 30-150 | -11.01 (3.53) |  | 1.96 (0.51) | 3.84 | 0.00 | -0.71 |  | 0.51 (0.03-0.78) | 0.67 (0.06-0.87) |  | 0.51 | 16.84 (12.96-24.07) |
|  |  | 30-180 | -10.36 (2.84) |  | 1.31 (0.38) | 3.44 | 0.00 | -0.56 |  | 0.62 (0.16-0.84) | 0.76 (0.28-0.91) |  | 0.38 | 12.98 (9.98-18.55) |
|  |  | 30-210 | -9.85 (2.61) |  | 0.8 (0.27) | 2.93 | 0.01 | -0.36 |  | 0.78 (0.46-0.91) | 0.88 (0.63-0.95) |  | 0.27 | 9.54 (7.34-13.64) |
|  |  | 30-240 | -9.6 (2.14) |  | 0.54 (0.19) | 2.79 | 0.01 | -0.28 |  | 0.86 (0.62-0.94) | 0.92 (0.76-0.97) |  | 0.19 | 6.89 (5.3-9.85) |
|  |  | 30-300 | -8.76 (1.83) |  | -0.38 (0.16) | -2.41 | 0.03 | 0.17 |  | 0.9 (0.73-0.96) | 0.95 (0.85-0.98) |  | 0.16 | 5.6 (4.26-8.18) |
|  |  | 60-90 | -13.22 (6.73) |  | 4.16 (1.31) | 3.17 | 0.00 | -0.85 |  | 0.16 (-0.15-0.49) | 0.27 (-0.36-0.66) |  | 1.31 | 39.13 (30.1-55.92) |
|  |  | 60-120 | -12.16 (4.65) |  | 3.11 (0.85) | 3.66 | 0.00 | -0.89 |  | 0.26 (-0.09-0.58) | 0.41 (-0.2-0.73) |  | 0.85 | 26.55 (20.43-37.94) |
|  |  | 60-150 | -11.95 (3.21) |  | 2.9 (0.43) | 6.73 | 0.00 | -1.14 |  | 0.42 (-0.11-0.76) | 0.59 (-0.24-0.86) |  | 0.43 | 13.6 (10.46-19.43) |
|  |  | 60-210 | -10.19 (2.49) |  | 1.13 (0.27) | 4.21 | 0.00 | -0.54 |  | 0.72 (0.18-0.9) | 0.84 (0.3-0.95) |  | 0.27 | 9.27 (7.13-13.25) |
|  |  | 60-240 | -9.83 (1.91) |  | 0.78 (0.17) | 4.59 | 0.00 | -0.43 |  | 0.83 (0.27-0.94) | 0.91 (0.43-0.97) |  | 0.17 | 5.95 (4.58-8.51) |
|  |  | 90-120 | -11.11 (6.92) |  | 2.05 (1.41) | 1.46 | 0.16 | -0.41 |  | 0.13 (-0.27-0.51) | 0.23 (-0.74-0.67) |  | 1.41 | 46.32 (35.64-66.2) |
|  |  | 90-150 | -11.32 (4.26) |  | 2.27 (0.75) | 3.02 | 0.01 | -0.70 |  | 0.33 (-0.04-0.64) | 0.5 (-0.09-0.78) |  | 0.75 | 24.47 (18.82-34.97) |
|  |  | 90-180 | -10.14 (2.86) |  | 1.08 (0.48) | 2.23 | 0.04 | -0.46 |  | 0.49 (0.11-0.75) | 0.66 (0.2-0.85) |  | 0.48 | 16.74 (12.88-23.92) |
|  |  | 90-210 | -9.43 (2.57) |  | 0.38 (0.35) | 1.08 | 0.29 | 0.58 |  | 0.71 (0.43-0.87) | 0.83 (0.6-0.93) |  | 0.35 | 12.53 (9.64-17.9) |
|  |  | 90-240 | -9.16 (1.99) |  | 0.1 (0.29) | 0.35 | 0.73 | -0.06 |  | 0.74 (0.47-0.88) | 0.85 (0.64-0.94) |  | 0.29 | 10.41 (8.01-14.87) |
|  |  | 90-300 | -8.15 (1.45) |  | -0.9 (0.21) | -4.38 | 0.00 | 0.58 |  | 0.7 (0.12-0.89) | 0.82 (0.22-0.94) |  | 0.21 | 7.96 (6.12-11.38) |
|  |  | 120-150 | -11.54 (7.47) |  | 2.49 (1.49) | 1.67 | 0.11 | -0.46 |  | 0.16 (-0.23-0.52) | 0.27 (-0.61-0.69) |  | 1.49 | 47.88 (36.84-68.43) |
|  |  | 120-180 | -9.65 (3.81) |  | 0.6 (0.74) | 0.81 | 0.43 | -0.20 |  | 0.3 (-0.13-0.64) | 0.47 (-0.29-0.78) |  | 0.74 | 26.26 (20.2-37.53) |
|  |  | 120-210 | -8.87 (2.8) |  | -0.18 (0.43) | -0.43 | 0.67 | 0.08 |  | 0.63 (0.29-0.83) | 0.77 (0.45-0.91) |  | 0.43 | 15.76 (12.12-22.52) |
|  |  | 120-240 | -8.67 (2.14) |  | -0.39 (0.37) | -1.06 | 0.30 | 0.20 |  | 0.6 (0.25-0.81) | 0.75 (0.4-0.89) |  | 0.37 | 13.7 (10.54-19.58) |
|  |  | 120-300 | -7.66 (1.53) |  | -1.39 (0.28) | -5.03 | 0.00 | 0.87 |  | 0.49 (-0.06-0.79) | 0.66 (-0.13-0.88) |  | 0.28 | 11.03 (8.48-15.76) |
|  |  | 150-180 | -7.76 (3.45) |  | -1.29 (0.8) | -1.61 | 0.12 | 0.48 |  | 0.03 (-0.35-0.42) | 0.06 (-1.07-0.59) |  | 0.80 | 31.69 (24.38-45.29) |
|  |  | 150-210 | -7.54 (2.81) |  | -1.52 (0.51) | -2.98 | 0.01 | 0.66 |  | 0.39 (0-0.69) | 0.56 (0-0.81) |  | 0.51 | 20.32 (15.63-29.04) |
|  |  | 150-240 | -7.71 (1.75) |  | -1.35 (0.38) | -3.50 | 0.00 | 0.79 |  | 0.35 (-0.05-0.66) | 0.51 (-0.1-0.79) |  | 0.38 | 15.2 (11.7-21.72) |
|  |  | 150-300 | -6.88 (1.16) |  | -2.17 (0.3) | -7.25 | 0.00 | 1.51 |  | 0.24 (-0.1-0.6) | 0.39 (-0.23-0.75) |  | 0.30 | 12.48 (9.6-17.83) |
|  |  | 180-210 | -7.31 (5.73) |  | -1.74 (1.09) | -1.60 | 0.13 | 0.41 |  | 0.25 (-0.15-0.59) | 0.4 (-0.35-0.74) |  | 1.09 | 44.24 (34.03-63.22) |
|  |  | 180-240 | -7.68 (2.63) |  | -0.55 (1.2) | -2.62 | 0.02 | 0.62 |  | 0.32 (-0.05-0.64) | 0.49 (-0.11-0.78) |  | 0.52 | 20.74 (15.96-29.64) |
|  |  | 180-300 | -6.66 (1.47) |  | -0.67 (0.43) | -7.29 | 0.00 | 1.53 |  | 0.24 (-0.1-0.6) | 0.39 (-0.23-0.75) |  | 0.33 | 13.85 (10.66-19.8) |
|  |  | 210-240 | -8.05 (3.13) |  | -0.73 (2.04) | -1.23 | 0.23 | 0.40 |  | -0.17 (-0.53-0.26) | -0.4 (-2.3-0.41) |  | 0.82 | 31.65 (24.35-45.23) |
|  |  | 210-300 | -6.45 (1.11) |  | -0.72 (0.43) | -6.33 | 0.00 | 1.85 |  | 0.02 (-0.1-0.24) | 0.05 (-0.23-0.38) |  | 0.41 | 17.63 (13.56-25.19) |
|  |  | 240-300 | -5.64 (1.51) |  | -1.11 (0.62) | -8.45 | 0.00 | 2.15 |  | 0.09 (-0.07-0.34) | 0.16 (-0.16-0.51) |  | 0.40 | 18.23 (14.03-26.05) |

**Table 3.** Excluded KE S_fv_ 2-point methods.

| **9-point method** | | **2-point methods** | |  | **Systematic error** | | | |  | **Intra-class correlation** | |  | **Within individual error** | |
| --- | --- | --- | --- | --- | --- | --- | --- | --- | --- | --- | --- | --- | --- | --- |
|  |  | **Combination** | **Mean (±SD)** |  | **Mean bias (±SD)** | **t** | **sig** | **ES** |  | **ICCs (95% CI)** | **ICCa (95% CI)** |  | **SEM** | **CV % (95% CI)** |
| **P_max_ (Wkg^-2/3^)** | **22.25 (5.33)** | 30-60 | 83.71 (366.28) |  | -61.46 (365.65) | -0.79 | 0.44 | 0.24 |  | 0 (-0.42-0.42) | 0.01 (-1.45-0.59) |  | 77.96 | 488.03 (375.47-697.43) |
|  |  | 30-90 | 26.75 (36.86) |  | -4.5 (35.33) | -0.60 | 0.56 | 0.17 |  | 0.1 (-0.34-0.5) | 0.19 (-1.03-0.67) |  | 7.53 | 101.97 (78.45-145.72) |
|  |  | 30-120 | 30.49 (37.75) |  | -8.24 (35.16) | -1.10 | 0.28 | 0.31 |  | 0.15 (-0.27-0.53) | 0.26 (-0.76-0.69) |  | 7.50 | 94.28 (72.54-134.74) |
|  |  | 30-150 | 20.69 (6.99) |  | 1.56 (2.61) | 2.82 | 0.01 | -0.25 |  | 0.89 (0.68-0.96) | 0.94 (0.81-0.98) |  | 0.56 | 8.58 (6.6-12.26) |
|  |  | 30-180 | 21.27 (6.3) |  | 0.99 (2.21) | 2.09 | 0.05 | -0.17 |  | 0.92 (0.8-0.97) | 0.96 (0.89-0.98) |  | 0.47 | 7.2 (5.54-10.29) |
|  |  | 30-300 | 22.18 (8.83) |  | 0.08 (5.68) | 0.06 | 0.95 | -0.01 |  | 0.71 (0.41-0.87) | 0.83 (0.58-0.93) |  | 1.21 | 18.09 (13.92-25.85) |
|  |  | 60-90 | -18.95 (125.27) |  | 41.2 (125.13) | 1.54 | 0.14 | -0.46 |  | 0 (-0.38-0.4) | 0.01 (-1.21-0.57) |  | 26.68 | 30.07 (22.87-43.92) |
|  |  | 60-120 | 20.86 (8.55) |  | 1.39 (6.84) | 0.95 | 0.35 | -0.19 |  | 0.54 (0.17-0.78) | 0.7 (0.29-0.88) |  | 1.46 | 22.44 (17.26-32.07) |
|  |  | 60-150 | 19.39 (5.48) |  | 2.86 (2.18) | 6.15 | 0.00 | -0.53 |  | 0.81 (0.04-0.95) | 0.89 (0.07-0.97) |  | 0.46 | 7.4 (5.7-10.58) |
|  |  | 60-210 | 21.4 (4.78) |  | 0.85 (1.14) | 3.50 | 0.00 | -0.17 |  | 0.96 (0.84-0.99) | 0.98 (0.91-0.99) |  | 0.24 | 3.71 (2.86-5.31) |
|  |  | 60-240 | 21.81 (4.87) |  | 0.44 (0.91) | 2.26 | 0.03 | -0.09 |  | 0.98 (0.95-0.99) | 0.99 (0.97-1) |  | 0.19 | 2.92 (2.25-4.17) |
|  |  | 60-300 | 23.73 (5.32) |  | -1.48 (0.62) | -11.26 | 0.00 | 0.28 |  | 0.96 (0.05-0.99) | 0.98 (0.1-1) |  | 0.13 | 1.9 (1.46-2.72) |
|  |  | 90-120 | 24.68 (34.63) |  | -2.43 (32.89) | -0.35 | 0.73 | 0.10 |  | 0.12 (-0.33-0.52) | 0.22 (-0.97-0.68) |  | 7.01 | 99.12 (76.26-141.65) |
|  |  | 90-150 | 20.5 (6.38) |  | 1.75 (3.39) | 2.43 | 0.02 | -0.30 |  | 0.80 (0.55-0.92) | 0.89 (0.71-0.96) |  | 0.72 | 11.2 (8.62-16.01) |
|  |  | 90-180 | 21.18 (5.91) |  | 1.07 (2.81) | 1.79 | 0.09 | -0.19 |  | 0.86 (0.70-0.94) | 0.93 (0.82-0.97) |  | 0.60 | 9.15 (7.04-13.08) |
|  |  | 90-300 | 23.67 (5.45) |  | -1.42 (0.67) | -9.97 | 0.00 | 0.26 |  | 0.96 (0.09-0.99) | 0.98 (0.17-1) |  | 0.14 | 2.06 (1.59-2.95) |
|  |  | 120-150 | 15.25 (23.41) |  | 7 (24.75) | 1.33 | 0.20 | -0.41 |  | -0.06 (-0.44-0.35) | -0.13 (-1.6-0.52) |  | 5.28 | 93.32 (71.8-133.36) |
|  |  | 120-180 | -15.21 (171.53) |  | 37.46 (172.29) | 1.02 | 0.32 | -0.15 |  | -0.01 (-0.42-0.41) | -0.02 (-1.44-0.58) |  | 36.73 | 12.38 (9.47-17.88) |
|  |  | 120-210 | 22.27 (5.42) |  | -0.02 (1.75) | -0.05 | 0.96 | 0.00 |  | 0.95 (0.88-0.98) | 0.97 (0.94-0.99) |  | 0.37 | 5.57 (4.29-7.96) |
|  |  | 120-240 | 22.29 (5.41) |  | -0.04 (1.37) | -0.14 | 0.89 | 0.01 |  | 0.97 (0.93-0.99) | 0.98 (0.96-0.99) |  | 0.29 | 4.36 (3.35-6.23) |
|  |  | 120-300 | 23.75 (5.67) |  | -1.5 (0.82) | -8.61 | 0.00 | 0.27 |  | 0.95 (0.14-0.99) | 0.98 (0.24-0.99) |  | 0.17 | 2.51 (1.93-3.59) |
|  |  | 150-180 | 33.28 (43.28) |  | -11.03 (44.21) | -1.17 | 0.26 | 0.36 |  | -0.03 (-0.43-0.39) | -0.06 (-1.49-0.56) |  | 9.43 | 112.59 (86.62-160.9) |
|  |  | 150-210 | 23.8 (5.46) |  | -1.55 (3.77) | -1.93 | 0.07 | 0.29 |  | 0.73 (0.46-0.88) | 0.85 (0.63-0.94) |  | 0.80 | 11.57 (8.9-16.54) |
|  |  | 150-240 | 22.57 (5.16) |  | -0.32 (1.33) | -1.12 | 0.28 | 0.06 |  | 0.97 (0.92-0.99) | 0.98 (0.96-0.99) |  | 0.28 | 4.20 (3.23-6) |
|  |  | 150-300 | 23.75 (5.66) |  | -1.5 (0.81) | -8.73 | 0.00 | 0.27 |  | 0.95 (0.13-0.99) | 0.98 (0.23-0.99) |  | 0.17 | 2.49 (1.91-3.55) |
|  |  | 180-210 | 10.55 (69.39) |  | 11.7 (69.39) | 0.79 | 0.44 | -0.24 |  | 0.01 (-0.42-0.42) | 0.01 (-1.44-0.59) |  | 14.79 | 68.18 (51.85-99.58) |
|  |  | 180-240 | 23.61 (7.7) |  | -1.36 (4.79) | -1.33 | 0.20 | 0.21 |  | 0.73 (0.46-0.88) | 0.85 (0.63-0.94) |  | 1.02 | 14.76 (11.35-21.09) |
|  |  | 180-300 | 23.96 (5.94) |  | -1.71 (1.22) | -6.60 | 0.00 | 0.30 |  | 0.94 (0.25-0.98) | 0.97 (0.4-0.99) |  | 0.26 | 3.72 (2.86-5.31) |
|  |  | 210-240 | 24.83 (9.02) |  | -2.58 (7.49) | -1.61 | 0.12 | 0.35 |  | 0.47 (0.09-0.74) | 0.64 (0.17-0.85) |  | 1.60 | 22.51 (17.32-32.18) |
|  |  | 210-300 | 24.03 (5.8) |  | -0.72 (0.43) | -7.63 | 0.00 | 0.32 |  | 0.93 (0.13-0.98) | 0.97 (0.24-0.99) |  | 0.23 | 3.35 (2.57-4.78) |
|  |  | 240-300 | 24.62 (6.24) |  | -1.11 (0.62) | -6.98 | 0.00 | 0.41 |  | 0.89 (0.08-0.97) | 0.94 (0.15-0.99) |  | 0.34 | 4.81 (3.7-6.88) |

**Table 4.** Excluded KE P_max_ 2-point methods.

| **9-point method** | | **2-point methods** | |  | **Systematic error** | | | |  | **Intra-class correlation** | |  | **Within individual error** | |
| --- | --- | --- | --- | --- | --- | --- | --- | --- | --- | --- | --- | --- | --- | --- |
|  |  | **Combination** | **Mean (±SD)** |  | **Mean bias (±SD)** | **t** | **sig** | **ES** |  | **ICCs (95% CI)** | **ICCa (95% CI)** |  | **SEM** | **CV % (95% CI)** |
| **F_0_ (Nkg^-2/3^)** | **15.01 (2.35)** | 30-60 | 15.21 (2.81) |  | -0.2 (2) | -0.47 | 0.64 | 0.08 |  | 0.71 (0.42-0.87) | 0.83 (0.59-0.93) |  | 0.43 | 9.35 (7.19-13.36) |
|  |  | 30-90 | 15.38 (2.59) |  | -0.37 (1.42) | -1.24 | 0.23 | 0.15 |  | 0.83 (0.64-0.93) | 0.91 (0.78-0.96) |  | 0.30 | 6.6 (5.08-9.43) |
|  |  | 30-120 | 15.44 (2.56) |  | -0.44 (1.19) | -1.72 | 0.10 | 0.18 |  | 0.87 (0.72-0.95) | 0.93 (0.83-0.97) |  | 0.25 | 5.55 (4.27-7.93) |
|  |  | 30-150 | 15.36 (2.52) |  | -0.36 (1.16) | -1.44 | 0.16 | 0.15 |  | 0.88 (0.74-0.95) | 0.94 (0.85-0.97) |  | 0.25 | 5.42 (4.17-7.75) |
|  |  | 60-90 | 15.9 (3.09) |  | -0.89 (1.54) | -2.71 | 0.01 | 0.32 |  | 0.81 (0.52-0.92) | 0.89 (0.69-0.96) |  | 0.33 | 7.06 (5.43-10.09) |
|  |  | 60-120 | 15.92 (2.96) |  | -0.91 (1.07) | -3.98 | 0.00 | 0.34 |  | 0.87 (0.48-0.96) | 0.93 (0.65-0.98) |  | 0.23 | 4.91 (3.78-7.01) |
|  |  | 60-150 | 15.62 (2.76) |  | -0.62 (0.85) | -3.40 | 0.00 | 0.24 |  | 0.92 (0.71-0.97) | 0.96 (0.83-0.99) |  | 0.18 | 3.93 (3.02-5.61) |
|  |  | 60-210 | 15.51 (2.56) |  | -0.5 (0.71) | -3.32 | 0.00 | 0.20 |  | 0.94 (0.78-0.98) | 0.97 (0.87-0.99) |  | 0.15 | 3.29 (2.53-4.71) |
|  |  | 60-240 | 15.37 (2.6) |  | -0.36 (0.63) | -2.69 | 0.01 | 0.15 |  | 0.96 (0.88-0.98) | 0.98 (0.93-0.99) |  | 0.13 | 2.94 (2.26-4.19) |
|  |  | 90-120 | 15.95 (3.72) |  | -0.95 (2.33) | -1.91 | 0.07 | 0.30 |  | 0.7 (0.4-0.86) | 0.82 (0.57-0.93) |  | 0.50 | 10.66 (8.2-15.23) |
|  |  | 120-150 | 14.15 (4.36) |  | 0.85 (3.94) | 1.01 | 0.32 | -0.24 |  | 0.37 (-0.05-0.68) | 0.54 (-0.1-0.81) |  | 0.84 | 19.09 (14.69-27.28) |
|  |  | 120-180 | 14.45 (2.41) |  | 0.56 (1.41) | 1.86 | 0.08 | -0.23 |  | 0.81 (0.59-0.92) | 0.9 (0.74-0.96) |  | 0.30 | 6.76 (5.2-9.66) |
|  |  | 120-210 | 14.56 (2.14) |  | 0.44 (1.45) | 1.44 | 0.16 | -0.20 |  | 0.79 (0.55-0.9) | 0.88 (0.71-0.95) |  | 0.31 | 6.93 (5.33-9.9) |
|  |  | 120-240 | 14.27 (2.17) |  | 0.74 (0.88) | 3.95 | 0.00 | -0.33 |  | 0.88 (0.51-0.96) | 0.94 (0.68-0.98) |  | 0.19 | 4.23 (3.25-6.04) |
|  |  | 120-300 | 14.19 (2.43) |  | 0.81 (1.06) | 3.58 | 0.00 | -0.34 |  | 0.86 (0.51-0.95) | 0.92 (0.67-0.97) |  | 0.23 | 5.16 (3.97-7.37) |
|  |  | 150-180 | 14.88 (5.99) |  | 0.12 (5.62) | 0.10 | 0.92 | -0.03 |  | 0.25 (-0.21-0.6) | 0.4 (-0.52-0.75) |  | 1.20 | 26.57 (20.44-37.97) |
|  |  | 150-210 | 14.91 (3.79) |  | 0.09 (3.57) | 0.12 | 0.91 | -0.03 |  | 0.37 (-0.07-0.68) | 0.54 (-0.14-0.81) |  | 0.76 | 16.87 (12.98-24.12) |
|  |  | 150-240 | 14.34 (3.12) |  | 0.66 (2.34) | 1.33 | 0.20 | -0.24 |  | 0.63 (0.31-0.83) | 0.78 (0.47-0.91) |  | 0.50 | 11.29 (8.69-16.13) |
|  |  | 150-300 | 14.21 (2.84) |  | 0.79 (1.62) | 2.30 | 0.03 | -0.30 |  | 0.78 (0.51-0.9) | 0.88 (0.67-0.95) |  | 0.35 | 7.85 (6.04-11.22) |
|  |  | 180-210 | 14.96 (4.44) |  | 0.05 (4.68) | 0.05 | 0.96 | -0.01 |  | 0.14 (-0.32-0.53) | 0.24 (-0.93-0.69) |  | 1.00 | 22.09 (16.99-31.57) |
|  |  | 180-240 | 13.91 (2.76) |  | 1.1 (1.85) | 2.77 | 0.01 | -0.43 |  | 0.68 (0.32-0.86) | 0.81 (0.48-0.93) |  | 0.39 | 9.06 (6.97-12.95) |
|  |  | 180-300 | 13.87 (3.45) |  | 1.13 (2.43) | 2.18 | 0.04 | -0.38 |  | 0.63 (0.28-0.83) | 0.77 (0.44-0.9) |  | 0.52 | 11.9 (9.16-17.01) |
|  |  | 210-240 | 12.51 (5.9) |  | 2.49 (4.71) | 2.49 | 0.02 | -0.56 |  | 0.4 (0.02-0.69) | 0.57 (0.03-0.82) |  | 1.00 | 24.19 (18.61-34.57) |
|  |  | 210-300 | 13.27 (4.84) |  | 1.74 (3.59) | 2.27 | 0.03 | -0.46 |  | 0.51 (0.14-0.76) | 0.68 (0.24-0.86) |  | 0.77 | 17.97 (13.82-25.67) |
|  |  | 240-300 | 13.81 (5.81) |  | 1.19 (4.98) | 1.12 | 0.27 | -0.27 |  | 0.37 (-0.04-0.68) | 0.54 (-0.09-0.81) |  | 1.06 | 24.42 (18.79-34.9) |

**Table 5** Excluded KF F_0_ 2-point methods.

| **9-point method** | | **2-point methods** | |  | **Systematic error** | | | |  | **Intra-class correlation** | |  | **Within individual error** | |
| --- | --- | --- | --- | --- | --- | --- | --- | --- | --- | --- | --- | --- | --- | --- |
|  |  | **Combination** | **Mean (±SD)** |  | **Mean bias (±SD)** | **t** | **sig** | **ES** |  | **ICCs (95% CI)** | **ICCa (95% CI)** |  | **SEM** | **CV % (95% CI)** |
| **v_0_ (ms^-1^)** | **3.36 (0.59)** | 30-60 | 1.63 (3.35) |  | 1.73 (3.52) | 2.31 | 0.03 | -0.72 |  | -0.06 (-0.37-0.32) | -0.13 (-1.2-0.48) |  | 0.75 | 99.89 (76.85-142.75) |
|  |  | 30-90 | 2.38 (3.72) |  | 0.98 (3.73) | 1.23 | 0.23 | -0.37 |  | 0.02 (-0.39-0.42) | 0.03 (-1.25-0.59) |  | 0.80 | 92.08 (70.84-131.59) |
|  |  | 30-120 | 3.65 (2.84) |  | -0.29 (2.66) | -0.51 | 0.61 | 0.14 |  | 0.16 (-0.28-0.54) | 0.28 (-0.79-0.7) |  | 0.57 | 53.67 (41.29-76.7) |
|  |  | 30-150 | 2.44 (4.97) |  | 0.92 (5.05) | 0.85 | 0.40 | -0.26 |  | -0.02 (-0.44-0.4) | -0.03 (-1.54-0.57) |  | 1.08 | 123.11 (94.72-175.94) |
|  |  | 30-180 | 3.36 (1.31) |  | 0 (1.11) | 0.01 | 0.99 | 0.00 |  | 0.41 (-0.01-0.71) | 0.59 (-0.03-0.83) |  | 0.24 | 23.37 (17.98-33.4) |
|  |  | 30-210 | 3.24 (1.04) |  | 0.11 (0.78) | 0.68 | 0.50 | -0.13 |  | 0.58 (0.22-0.8) | 0.73 (0.36-0.89) |  | 0.17 | 16.69 (12.84-23.85) |
|  |  | 30-240 | 3.42 (0.87) |  | -0.06 (0.57) | -0.51 | 0.62 | 0.08 |  | 0.71 (0.42-0.87) | 0.83 (0.59-0.93) |  | 0.12 | 11.93 (9.17-17.04) |
|  |  | 30-300 | 3.53 (0.7) |  | -0.17 (0.28) | -2.78 | 0.01 | 0.26 |  | 0.88 (0.66-0.95) | 0.93 (0.79-0.98) |  | 0.06 | 5.86 (4.51-8.37) |
|  |  | 60-90 | 2.51 (2.23) |  | 0.85 (2.14) | 1.86 | 0.08 | -0.52 |  | 0.13 (-0.25-0.49) | 0.22 (-0.67-0.66) |  | 0.46 | 51.63 (39.72-73.78) |
|  |  | 60-120 | 4.42 (8.48) |  | -1.06 (8.53) | -0.58 | 0.57 | 0.18 |  | -0.01 (-0.44-0.41) | -0.02 (-1.56-0.59) |  | 1.82 | 155.08 (119.31-221.61) |
|  |  | 60-150 | 3.16 (1.15) |  | 0.2 (0.83) | 1.14 | 0.27 | -0.22 |  | 0.58 (0.23-0.8) | 0.74 (0.38-0.89) |  | 0.18 | 18.1 (13.92-25.86) |
|  |  | 60-180 | 3.07 (0.68) |  | 0.29 (0.56) | 2.41 | 0.03 | -0.45 |  | 0.57 (0.2-0.8) | 0.73 (0.34-0.89) |  | 0.12 | 12.24 (9.42-17.49) |
|  |  | 60-210 | 3.11 (0.77) |  | 0.25 (0.51) | 2.29 | 0.03 | -0.36 |  | 0.69 (0.36-0.85) | 0.81 (0.53-0.92) |  | 0.11 | 11.18 (8.6-15.97) |
|  |  | 60-240 | 3.3 (0.6) |  | 0.06 (0.33) | 0.81 | 0.43 | 0.26 |  | 0.85 (0.68-0.94) | 0.92 (0.81-0.97) |  | 0.07 | 5.86 (4.51-8.37) |
|  |  | 60-300 | 3.51 (0.63) |  | -0.15 (0.34) | -2.11 | 0.05 | 0.25 |  | 0.83 (0.61-0.93) | 0.91 (0.76-0.96) |  | 0.07 | 6.97 (5.36-9.96) |
|  |  | 90-120 | 2.78 (4.28) |  | 0.57 (4.26) | 0.63 | 0.53 | -0.19 |  | 0.03 (-0.41-0.44) | 0.05 (-1.37-0.61) |  | 0.91 | 98.05 (75.44-140.12) |
|  |  | 90-150 | 3.56 (1.7) |  | -0.2 (1.34) | -0.70 | 0.49 | 0.16 |  | 0.45 (0.04-0.73) | 0.62 (0.08-0.84) |  | 0.29 | 27.45 (21.12-39.22) |
|  |  | 90-180 | 3.28 (0.93) |  | 0.08 (0.81) | 0.47 | 0.65 | -0.10 |  | 0.46 (0.05-0.74) | 0.63 (0.1-0.85) |  | 0.17 | 17.36 (13.36-24.82) |
|  |  | 90-210 | 3.29 (0.94) |  | 0.07 (0.71) | 0.44 | 0.67 | -0.08 |  | 0.61 (0.25-0.82) | 0.76 (0.4-0.9) |  | 0.15 | 15.03 (11.56-21.48) |
|  |  | 90-240 | 3.47 (0.69) |  | -0.11 (0.43) | -1.19 | 0.25 | 0.17 |  | 0.77 (0.54-0.9) | 0.87 (0.7-0.95) |  | 0.09 | 8.86 (6.81-12.66) |
|  |  | 90-300 | 3.64 (0.68) |  | -0.29 (0.4) | -3.39 | 0.00 | 0.45 |  | 0.74 (0.32-0.9) | 0.85 (0.49-0.95) |  | 0.08 | 8.02 (6.17-11.46) |
|  |  | 120-150 | 5.41 (31.67) |  | -2.05 (31.55) | -0.31 | 0.76 | 0.09 |  | 0.01 (-0.43-0.43) | 0.02 (-1.52-0.6) |  | 6.73 | 508.87 (391.5-727.2) |
|  |  | 120-180 | 3.83 (1.36) |  | -0.47 (1.25) | -1.77 | 0.09 | 0.45 |  | 0.27 (-0.12-0.6) | 0.43 (-0.28-0.75) |  | 0.27 | 24.59 (18.92-35.14) |
|  |  | 120-210 | 3.63 (1.19) |  | -0.27 (0.94) | -1.34 | 0.19 | 0.29 |  | 0.49 (0.11-0.75) | 0.66 (0.2-0.86) |  | 0.20 | 19.05 (14.65-27.22) |
|  |  | 120-240 | 3.71 (0.77) |  | -0.35 (0.5) | -3.31 | 0.00 | 0.51 |  | 0.66 (0.22-0.86) | 0.79 (0.36-0.92) |  | 0.11 | 9.98 (7.68-14.26) |
|  |  | 120-300 | 3.84 (0.76) |  | -0.48 (0.46) | -4.89 | 0.00 | 0.71 |  | 0.62 (0.01-0.86) | 0.77 (0.01-0.92) |  | 0.10 | 9.11 (7.01-13.02) |
|  |  | 150-180 | 9.06 (26.39) |  | -5.7 (26.55) | -1.01 | 0.33 | 0.31 |  | -0.01 (-0.42-0.4) | -0.02 (-1.46-0.58) |  | 5.66 | 302.32 (232.59-432.04) |
|  |  | 150-210 | 3.28 (2.15) |  | 0.08 (2.12) | 0.12 | 0.91 | -0.03 |  | 0.37 (-0.07-0.68) | 0.54 (-0.14-0.81) |  | 0.76 | 16.87 (12.98-24.12) |
|  |  | 150-240 | 2.39 (5.8) |  | 0.97 (5.8) | 1.33 | 0.20 | -0.24 |  | 0.63 (0.31-0.83) | 0.78 (0.47-0.91) |  | 0.50 | 11.29 (8.69-16.13) |
|  |  | 150-300 | 3.88 (0.8) |  | -0.53 (0.73) | 2.30 | 0.03 | -0.30 |  | 0.78 (0.51-0.9) | 0.88 (0.67-0.95) |  | 0.35 | 7.85 (6.04-11.22) |
|  |  | 180-210 | 4.15 (4.45) |  | -0.88 (4.32) | 0.05 | 0.96 | -0.01 |  | 0.14 (-0.32-0.53) | 0.24 (-0.93-0.69) |  | 1.00 | 22.09 (16.99-31.57) |
|  |  | 180-240 | 4.15 (1.46) |  | -0.8 (1.31) | 2.77 | 0.01 | -0.43 |  | 0.68 (0.32-0.86) | 0.81 (0.48-0.93) |  | 0.39 | 9.06 (6.97-12.95) |
|  |  | 180-300 | 4.17 (1.08) |  | -0.82 (0.91) | 2.18 | 0.04 | -0.38 |  | 0.63 (0.28-0.83) | 0.77 (0.44-0.9) |  | 0.52 | 11.9 (9.16-17.01) |
|  |  | 210-240 | 2.59 (4.29) |  | 0.77 (4.27) | 2.49 | 0.02 | -0.56 |  | 0.4 (0.02-0.69) | 0.57 (0.03-0.82) |  | 1.00 | 24.19 (18.61-34.57) |
|  |  | 210-300 | 1.24 (22.24) |  | 2.12 (22.14) | 2.27 | 0.03 | -0.46 |  | 0.51 (0.14-0.76) | 0.68 (0.24-0.86) |  | 0.77 | 17.97 (13.82-25.67) |
|  |  | 240-300 | 7.48 (10.65) |  | -4.12 (10.8) | 1.12 | 0.27 | -0.27 |  | 0.37 (-0.04-0.68) | 0.54 (-0.09-0.81) |  | 1.06 | 24.42 (18.79-34.9) |

**Table 6.** Excluded KF v_0_ 2-point methods.

| **9-point method** | | **2-point methods** | |  | **Systematic error** | | | |  | **Intra-class correlation** | |  | **Within individual error** | |
| --- | --- | --- | --- | --- | --- | --- | --- | --- | --- | --- | --- | --- | --- | --- |
|  |  | **Combination** | **Mean (±SD)** |  | **Mean bias (±SD)** | **t** | **sig** | **ES** |  | **ICCs (95% CI)** | **ICCa (95% CI)** |  | **SEM** | **CV % (95% CI)** |
| **S_fv_ (Nms^-1^kg^-2/3^)** | **-4.54 (0.74)** | 30-60 | -4.37 (6.63) |  | -0.18 (6.46) | -0.13 | 0.90 | 0.04 |  | 0.07 (-0.38-0.47) | 0.12 (-1.24-0.64) |  | 1.38 | 102.54 (78.89-146.54) |
|  |  | 30-90 | -5.36 (3.5) |  | 0.82 (3.25) | 1.18 | 0.25 | -0.32 |  | 0.17 (-0.25-0.54) | 0.29 (-0.65-0.7) |  | 0.69 | 46.38 (35.68-66.28) |
|  |  | 30-120 | -5.68 (2.41) |  | 1.14 (2.03) | 2.63 | 0.02 | -0.64 |  | 0.3 (-0.07-0.62) | 0.46 (-0.15-0.76) |  | 0.43 | 28.15 (21.66-40.23) |
|  |  | 30-150 | -5.22 (2.29) |  | 0.68 (1.88) | 1.70 | 0.10 | -0.40 |  | 0.37 (-0.02-0.67) | 0.54 (-0.04-0.81) |  | 0.40 | 27.22 (20.95-38.91) |
|  |  | 30-180 | -5.09 (1.7) |  | 0.55 (1.36) | 1.90 | 0.07 | -0.42 |  | 0.43 (0.05-0.71) | 0.6 (0.1-0.83) |  | 0.29 | 19.94 (15.34-28.5) |
|  |  | 30-210 | -5.04 (1.27) |  | 0.5 (0.99) | 2.34 | 0.03 | -0.48 |  | 0.5 (0.12-0.75) | 0.66 (0.21-0.86) |  | 0.21 | 14.66 (11.28-20.95) |
|  |  | 30-240 | -4.7 (1.2) |  | 0.16 (0.82) | 0.91 | 0.37 | -0.16 |  | 0.66 (0.35-0.84) | 0.8 (0.52-0.92) |  | 0.17 | 12.58 (9.68-17.98) |
|  |  | 30-300 | -4.44 (0.94) |  | -0.1 (0.43) | -1.06 | 0.30 | 0.12 |  | 0.87 (0.72-0.94) | 0.93 (0.84-0.97) |  | 0.09 | 6.76 (5.2-9.66) |
|  |  | 60-90 | -6.36 (3.55) |  | 1.82 (3.38) | 2.52 | 0.02 | -0.71 |  | 0.11 (-0.22-0.46) | 0.19 (-0.57-0.63) |  | 0.72 | 43.86 (33.74-62.68) |
|  |  | 60-120 | -6.34 (2.25) |  | 1.8 (1.92) | 4.41 | 0.00 | -1.11 |  | 0.23 (-0.1-0.55) | 0.37 (-0.23-0.71) |  | 0.41 | 24.67 (18.98-35.26) |
|  |  | 60-150 | -5.51 (1.97) |  | 0.96 (1.53) | 2.96 | 0.01 | -0.65 |  | 0.4 (0.01-0.69) | 0.57 (0.02-0.82) |  | 0.33 | 21.48 (16.53-30.7) |
|  |  | 60-180 | -5.27 (1.32) |  | 0.73 (1.01) | 3.39 | 0.00 | -0.68 |  | 0.46 (0.03-0.74) | 0.63 (0.06-0.85) |  | 0.22 | 14.55 (11.19-20.79) |
|  |  | 60-210 | -5.17 (1.08) |  | 0.63 (0.93) | 3.18 | 0.00 | -0.68 |  | 0.41 (0.01-0.7) | 0.58 (0.01-0.83) |  | 0.20 | 13.55 (10.43-19.37) |
|  |  | 60-240 | -4.76 (0.93) |  | 0.21 (0.58) | 1.73 | 0.10 | -0.25 |  | 0.75 (0.48-0.89) | 0.85 (0.65-0.94) |  | 0.12 | 8.82 (6.79-12.61) |
|  |  | 60-300 | -4.45 (0.97) |  | -0.09 (0.65) | -0.62 | 0.54 | 0.10 |  | 0.72 (0.44-0.87) | 0.84 (0.61-0.93) |  | 0.14 | 10.28 (7.91-14.69) |
|  |  | 90-120 | -6.33 (3.87) |  | 1.79 (3.65) | 2.30 | 0.03 | -0.64 |  | 0.12 (-0.23-0.48) | 0.22 (-0.58-0.65) |  | 0.78 | 47.4 (36.47-67.74) |
|  |  | 90-150 | -5.08 (2.21) |  | 0.54 (1.77) | 1.43 | 0.17 | -0.33 |  | 0.41 (0.02-0.7) | 0.59 (0.04-0.82) |  | 0.38 | 26.01 (20.01-37.16) |
|  |  | 90-180 | -4.91 (1.42) |  | 0.37 (1.19) | 1.45 | 0.16 | -0.33 |  | 0.43 (0.04-0.71) | 0.6 (0.08-0.83) |  | 0.25 | 17.83 (13.72-25.48) |
|  |  | 90-210 | -4.88 (1.24) |  | 0.34 (1.2) | 1.31 | 0.20 | -0.33 |  | 0.3 (-0.11-0.63) | 0.46 (-0.24-0.77) |  | 0.26 | 17.97 (13.82-25.68) |
|  |  | 90-240 | -4.44 (0.98) |  | -0.11 (0.74) | -0.67 | 0.51 | 0.12 |  | 0.64 (0.31-0.83) | 0.78 (0.47-0.91) |  | 0.16 | 11.69 (8.99-16.7) |
|  |  | 90-300 | -4.18 (1) |  | -0.36 (0.77) | -2.20 | 0.04 | 0.41 |  | 0.59 (0.23-0.8) | 0.74 (0.37-0.89) |  | 0.16 | 12.4 (9.54-17.72) |
|  |  | 120-150 | -3.83 (5.12) |  | -0.71 (4.93) | -0.67 | 0.51 | 0.19 |  | 0.09 (-0.35-0.49) | 0.17 (-1.05-0.66) |  | 1.05 | 83.22 (64.02-118.92) |
|  |  | 120-180 | -4.2 (1.6) |  | -0.34 (1.58) | -1.01 | 0.32 | 0.27 |  | 0.2 (-0.23-0.56) | 0.33 (-0.61-0.72) |  | 0.34 | 25.56 (19.67-36.53) |
|  |  | 120-210 | -4.39 (1.39) |  | -0.15 (1.54) | -0.45 | 0.66 | 0.13 |  | 0.05 (-0.39-0.46) | 0.1 (-1.28-0.63) |  | 0.33 | 24.29 (18.69-34.71) |
|  |  | 120-240 | -3.96 (0.8) |  | -0.58 (0.74) | -3.67 | 0.00 | 0.75 |  | 0.43 (-0.01-0.72) | 0.6 (-0.01-0.84) |  | 0.16 | 12.31 (9.47-17.59) |
|  |  | 120-300 | -3.83 (1.08) |  | -0.72 (0.97) | -3.47 | 0.00 | 0.78 |  | 0.35 (-0.04-0.66) | 0.52 (-0.08-0.8) |  | 0.21 | 16.41 (12.63-23.46) |
|  |  | 150-180 | -4.57 (5.21) |  | 0.03 (5.38) | 0.02 | 0.98 | -0.01 |  | -0.05 (-0.48-0.39) | -0.1 (-1.85-0.56) |  | 1.15 | 83.51 (64.25-119.34) |
|  |  | 150-210 | -4.67 (2.89) |  | 0.13 (3.15) | 0.19 | 0.85 | -0.06 |  | -0.12 (-0.54-0.32) | -0.28 (-2.36-0.49) |  | 0.67 | 48.32 (37.18-69.06) |
|  |  | 150-240 | -4.01 (1.65) |  | -0.54 (1.8) | -1.40 | 0.18 | 0.42 |  | 0.01 (-0.38-0.41) | 0.02 (-1.22-0.58) |  | 0.38 | 29.69 (22.84-42.43) |
|  |  | 150-300 | -3.82 (1.15) |  | -0.72 (1.2) | -2.81 | 0.01 | 0.74 |  | 0.19 (-0.15-0.52) | 0.31 (-0.35-0.69) |  | 0.26 | 20.28 (15.61-28.99) |
|  |  | 180-210 | -4.78 (3.87) |  | 0.23 (4.05) | 0.27 | 0.79 | -0.08 |  | -0.06 (-0.49-0.38) | -0.12 (-1.9-0.55) |  | 0.86 | 61.42 (47.26-87.78) |
|  |  | 180-240 | -3.72 (1.39) |  | -0.82 (1.35) | -2.84 | 0.01 | 0.73 |  | 0.22 (-0.13-0.55) | 0.36 (-0.29-0.71) |  | 0.29 | 23.07 (17.75-32.97) |
|  |  | 180-300 | -3.64 (1.83) |  | -0.9 (1.75) | -2.42 | 0.02 | 0.65 |  | 0.18 (-0.17-0.53) | 0.31 (-0.4-0.69) |  | 0.37 | 30.29 (23.3-43.28) |
|  |  | 210-240 | -2.67 (3.59) |  | -1.87 (3.35) | -2.61 | 0.02 | 0.72 |  | 0.13 (-0.2-0.48) | 0.24 (-0.49-0.65) |  | 0.71 | 65.72 (50.56-93.92) |
|  |  | 210-300 | -3.26 (2.57) |  | -1.28 (2.4) | -2.51 | 0.02 | 0.68 |  | 0.17 (-0.18-0.51) | 0.28 (-0.43-0.68) |  | 0.51 | 43.48 (33.45-62.14) |
|  |  | 240-300 | -3.55 (3.25) |  | -0.99 (3.18) | -1.46 | 0.16 | 0.42 |  | 0.09 (-0.31-0.47) | 0.16 (-0.89-0.64) |  | 0.68 | 55.58 (42.76-79.43) |

**Table 7.** Excluded KF S_fv_ 2-point methods.

| **9-point method** | | **2-point methods** | |  | **Systematic error** | | | |  | **Intra-class correlation** | |  | **Within individual error** | |
| --- | --- | --- | --- | --- | --- | --- | --- | --- | --- | --- | --- | --- | --- | --- |
|  |  | **Combination** | **Mean (±SD)** |  | **Mean bias (±SD)** | **t** | **sig** | **ES** |  | **ICCs (95% CI)** | **ICCa (95% CI)** |  | **SEM** | **CV % (95% CI)** |
| **P_max_ (Wkg^-2/3^)** | **12.72 (3.46)** | 30-60 | 6.59 (12.48) |  | 6.13 (13.21) | 2.18 | 0.04 | -0.67 |  | -0.03 (-0.36-0.35) | -0.07 (-1.13-0.51) |  | 2.97 | 96.74 (74.43-138.25) |
|  |  | 30-90 | 9.84 (13.16) |  | 2.88 (12.68) | 1.07 | 0.30 | -0.30 |  | 0.13 (-0.29-0.51) | 0.23 (-0.82-0.68) |  | 10.50 | 79.47 (61.14-113.56) |
|  |  | 30-120 | 13.61 (9.39) |  | -0.88 (8.18) | -0.51 | 0.62 | 0.12 |  | 0.34 (-0.1-0.66) | 0.51 (-0.21-0.8) |  | 1.78 | 43.92 (33.79-62.77) |
|  |  | 30-150 | 9.2 (17.28) |  | 3.53 (18.03) | 0.92 | 0.37 | -0.28 |  | -0.05 (-0.46-0.38) | -0.1 (-1.68-0.55) |  | 4.59 | 34.47 (26.37-49.78) |
|  |  | 30-180 | 12.83 (5.21) |  | -0.11 (3.47) | -0.14 | 0.89 | 0.02 |  | 0.7 (0.4-0.87) | 0.83 (0.57-0.93) |  | 0.90 | 19.2 (14.77-27.43) |
|  |  | 30-210 | 12.54 (4.62) |  | 0.18 (2.43) | 0.35 | 0.73 | -0.04 |  | 0.83 (0.63-0.93) | 0.91 (0.77-0.96) |  | 1.07 | 13.61 (10.47-19.45) |
|  |  | 30-240 | 13.08 (3.85) |  | -0.36 (1.39) | -1.22 | 0.24 | 0.10 |  | 0.93 (0.83-0.97) | 0.96 (0.91-0.98) |  | 1.10 | 7.62 (5.86-10.88) |
|  |  | 30-300 | 13.49 (3.62) |  | -0.77 (1.19) | -3.05 | 0.01 | 0.22 |  | 0.92 (0.75-0.97) | 0.96 (0.86-0.99) |  | 1.72 | 6.4 (4.92-9.14) |
|  |  | 60-90 | 10.42 (6.68) |  | 2.3 (5.45) | 1.98 | 0.06 | -0.43 |  | 0.45 (0.07-0.72) | 0.62 (0.12-0.84) |  | 8.97 | 33.3 (25.62-47.59) |
|  |  | 60-120 | 14.5 (18.73) |  | -1.78 (19.54) | -0.43 | 0.67 | 0.13 |  | -0.06 (-0.48-0.38) | -0.12 (-1.86-0.55) |  | 4.17 | 101.52 (78.1-145.08) |
|  |  | 60-150 | 12.27 (5.04) |  | 0.45 (3.17) | 0.67 | 0.51 | -0.10 |  | 0.74 (0.47-0.88) | 0.85 (0.64-0.94) |  | 7.06 | 17.93 (13.79-25.62) |
|  |  | 60-210 | 12.17 (3.91) |  | 0.55 (1.7) | 1.51 | 0.15 | -0.15 |  | 0.89 (0.75-0.95) | 0.94 (0.86-0.98) |  | 3.18 | 9.66 (7.43-13.8) |
|  |  | 60-300 | 13.44 (3.5) |  | -0.72 (1) | -3.38 | 0.00 | 0.01 |  | 0.94 (0.77-0.98) | 0.97 (0.87-0.99) |  | 1.37 | 5.62 (4.32-8.03) |
|  |  | 90-120 | 10.32 (12.48) |  | 2.4 (12.9) | 0.87 | 0.39 | 0.21 |  | 0.01 (-0.41-0.42) | 0.02 (-1.4-0.59) |  | 2.89 | 5.4 (4.15-7.72) |
|  |  | 90-150 | 13.51 (7.03) |  | -0.79 (5.07) | -0.73 | 0.47 | -0.26 |  | 0.59 (0.23-0.8) | 0.86 (0.66-0.94) |  | 1.19 | 79.14 (60.89-113.09) |
|  |  | 90-180 | 12.48 (4.22) |  | 0.24 (2.74) | 0.41 | 0.69 | -0.06 |  | 0.76 (0.5-0.89) | 0.92 (0.81-0.97) |  | 0.69 | 15.38 (11.83-21.97) |
|  |  | 90-210 | 12.57 (4.27) |  | 0.15 (2.14) | 0.34 | 0.74 | -0.04 |  | 0.85 (0.68-0.94) | 0.97 (0.93-0.99) |  | 0.49 | 11.97 (9.21-17.11) |
|  |  | 90-300 | 13.54 (3.56) |  | -0.82 (1.06) | -3.61 | 0.00 | 0.23 |  | 0.93 (0.71-0.98) | 0.03 (-1.46-0.61) |  | 1.72 | 5.71 (4.4-8.16) |
|  |  | 120-150 | 20.96 (91.1) |  | -8.24 (90.45) | -0.43 | 0.67 | 0.13 |  | 0.02 (-0.42-0.44) | 0.79 (0.5-0.91) |  | 19.39 | 379.78 (292.18-542.72) |
|  |  | 120-180 | 13.8 (5.46) |  | -1.08 (3.79) | -1.34 | 0.19 | 0.24 |  | 0.65 (0.33-0.84) | 0.9 (0.76-0.96) |  | 12.89 | 20.18 (15.53-28.84) |
|  |  | 120-210 | 13.23 (4.84) |  | -0.51 (2.54) | -0.94 | 0.36 | 0.12 |  | 0.82 (0.62-0.92) | 0.96 (0.9-0.99) |  | 4.01 | 13.87 (10.67-19.82) |
|  |  | 120-240 | 13.36 (3.84) |  | -0.64 (1.27) | -2.35 | 0.03 | 0.17 |  | 0.93 (0.81-0.97) | 0.96 (0.78-0.99) |  | 2.30 | 6.86 (5.28-9.81) |
|  |  | 120-300 | 13.67 (3.74) |  | -0.95 (1.13) | -3.95 | 0.00 | 0.26 |  | 0.92 (0.63-0.97) | -0.05 (-1.52-0.57) |  | 1.73 | 6.05 (4.66-8.65) |
|  |  | 150-180 | 24.95 (56.24) |  | -12.23 (56.96) | -1.01 | 0.33 | 0.31 |  | -0.02 (-0.43-0.39) | 0.6 (0.02-0.84) |  | 12.15 | 213.84 (164.52-305.59) |
|  |  | 150-210 | 12.3 (6.54) |  | 0.42 (5.64) | 0.35 | 0.73 | -0.08 |  | 0.43 (0.01-0.72) | 0.07 (-1.26-0.62) |  | 1.26 | 31.87 (24.52-45.54) |
|  |  | 150-240 | 9.73 (16.03) |  | 2.99 (16.09) | 0.87 | 0.39 | -0.26 |  | 0.04 (-0.39-0.45) | 0.94 (0.77-0.98) |  | 3.51 | 101.36 (77.98-144.85) |
|  |  | 150-300 | 13.74 (3.7) |  | -1.02 (1.45) | -3.28 | 0.00 | 0.28 |  | 0.89 (0.62-0.96) | 0.35 (-0.6-0.73) |  | 1.74 | 7.76 (5.97-11.09) |
|  |  | 180-210 | 14.42 (13.79) |  | -1.7 (12.66) | -0.63 | 0.54 | 0.17 |  | 0.21 (-0.23-0.58) | 0.86 (0.61-0.94) |  | 2.85 | 65.96 (50.75-94.27) |
|  |  | 180-240 | 14.21 (4.7) |  | -1.49 (2.67) | -2.62 | 0.02 | 0.36 |  | 0.75 (0.43-0.89) | 0.92 (0.48-0.98) |  | 1.30 | 14 (10.77-20.01) |
|  |  | 180-300 | 14.18 (3.91) |  | -1.46 (1.45) | -4.72 | 0.00 | 0.40 |  | 0.86 (0.31-0.96) | 0.3 (-0.63-0.71) |  | 1.77 | 7.65 (5.88-10.93) |
|  |  | 210-240 | 10.18 (10.36) |  | 2.54 (9.89) | 1.21 | 0.24 | -0.33 |  | 0.18 (-0.24-0.55) | 0.17 (-1.12-0.66) |  | 2.79 | 61.05 (46.97-87.24) |
|  |  | 210-300 | 11.56 (33.47) |  | 1.16 (32.13) | 0.17 | 0.87 | -0.05 |  | 0.09 (-0.36-0.5) | -0.03 (-1.19-0.55) |  | 7.03 | 187.08 (143.93-267.35) |
|  |  | 240-300 | 20.49 (19.55) |  | -7.77 (20.02) | -1.82 | 0.08 | 0.55 |  | -0.02 (-0.37-0.38) | 0.74 (0.37-0.89) |  | 4.71 | 85.24 (65.58-121.81) |

**Table 8.** Excluded KF P_max_ 2-point methods
